# Brain topology alteration in Alzheimer’s disease brain networks: A multi-center study

**DOI:** 10.1016/j.nicl.2025.103919

**Published:** 2025-11-30

**Authors:** Longhao Ma, Pan Wang, Dawei Wang, Hongxiang Yao, Bo Zhou, Yonghua Zhao, Zhengluan Liao, Yan Chen, Xi Zhang, Ying Han, Jie Lu, Kun Zhao, Yihe Zhang, Yong Liu, for the Alzheimer’s Disease Neuroimaging Initiative, for the Multi-Center Alzheimer’s Disease Imaging (MCADI) Consortium

**Affiliations:** a School of Artificial Intelligence, Beijing University of Posts and Telecommunications, Beijing, China; b Queen Mary School Hainan, Beijing University of Posts and Telecommunications, Hainan, China; c Department of Neurology, Tianjin Huanhu Hospital, Tianjin, China; d Department of Radiology, Qilu Hospital of Shandong University, Ji’nan, China; e Department of Radiology, the Second Medical Centre, National Clinical Research Centre for Geriatric Diseases, Chinese PLA General Hospital, Beijing, China; f Department of Neurology, the Second Medical Centre, National Clinical Research Centre for Geriatric Diseases, Chinese PLA General Hospital, Beijing, China; g The State Key Laboratory of Mechanism and Quality of Chinese Medicine, Institute of Chinese Medical Sciences, University of Macau, Taipa, Macao Special Administrative Region of China; h Department of Psychiatry, People’s Hospital of Hangzhou Medical College, Zhejiang Provincial People’s Hospital, Hangzhou, China; i State Key Laboratory of Digital Medical Engineering, Key Laboratory of Biomedical Engineering of Hainan Province, School of Biomedical Engineering, Hainan University, Sanya, Hainan, China; j National Clinical Research Centre for Geriatric Disorders, Beijing, China; k Centre of Alzheimer’s Disease, Beijing Institute for Brain Disorders, Beijing, China; l Department of Radiology, Xuanwu Hospital of Capital Medical University, Beijing, China; m Center for Inspur-BUPT, Beijing University of Posts and Telecommunications, Beijing, China; n Center for Inspur-BUPT, Shandong Key Laboratory of Advanced Computing, Inspur Computer Technology Co., Ltd, Ji’nan, China

**Keywords:** Alzheimer’s disease, Brain functional network, Centrality, Multicenter, Resting-state fMRI

## Abstract

Alterations in brain network centrality are key features of Alzheimer’s disease (AD) and may offer insights into the disruption of network organization underlying cognitive decline. We introduce a novel centrality metric, DomiRank, to characterize dominance-driven connectivity patterns in the human brain network, using a multi-center MRI dataset comprising 809 participants. Compared with conventional metrics, DomiRank centrality showed greater sensitivity in detecting AD-related network disruptions, particularly within the cingulate gyrus, precuneus, and subcortical hubs such as the basal ganglia—regions critical for cognition. Regional DomiRank alterations were significantly correlated with clinical cognitive scores, indicating their potential relevance to disease severity. Gene enrichment analysis revealed that areas with reduced DomiRank centrality were enriched for genes involved in synaptic signaling and neuronal communication, suggesting molecular mechanisms underlying network vulnerability. These findings highlight DomiRank centrality as a promising biomarker for characterizing network disorganization in AD, linking changes in brain connectivity with underlying molecular processes.

## Highlights

• Developed and validated DomiRank centrality, a novel graph metric integrating local/global connectivity to quantify hub dominance and detect subtle network vulnerabilities.
• In an 809-participant multi-center rs-fMRI cohort (HC/MCI/AD), DomiRank identified robust, reproducible brain network topology alterations.
• DomiRank showed strong associations with MMSE/MoCA scores, connecting network disruptions to clinically relevant cognitive decline trajectories.
• Cross-site validation confirmed DomiRank’s robustness/generalizability, outperforming traditional metrics in network robustness and patient classification.
• Gene enrichment analyses linked altered DomiRank hubs to synaptic signaling, iron transport, and neuroinflammatory pathways, bridging macroscale topology and AD molecular mechanisms.

## 1 Introduction

Alzheimer's disease (AD) is the most common neurodegenerative disease and the leading cause of dementia worldwide, thereby imposing a significant burden on public health (Alzheimer's Association, 2024, Braak and Braak, 1991, Scheltens et al., 2016). Despite decades of research, only a few disease-modifying therapies for AD have now been approved, yet their widespread clinical application remains limited due to poor efficacy in later stages, high costs, and intensive monitoring requirements (Smith et al., 2025, van Dyck et al., 2023, Wang et al., 2025, Zimmer et al., 2025). Given that AD progresses insidiously, with functional and structural brain changes occurring years before the onset of clinical symptoms, the limited effectiveness of late-stage interventions underscores the urgent need for early detection (Prescott et al., 2022, Ridha et al., 2006, Tondelli et al., 2012, Xie et al., 2020). Therefore, a deep understanding of the complex pathophysiology and the development of reliable biomarkers is urgently needed for the early detection of AD (Counts et al., 2017, Dubois et al., 2023).

Graph-theoretic approaches have revolutionized the analysis of the human brain by modeling it as a complex network, with nodes corresponding to anatomical or functional regions and edges representing structural or functional connections between them. (reflecting structural or functional connections) (Bassett and Bullmore, 2017, Bullmore and Sporns, 2009, Faskowitz et al., 2022, He and Evans, 2010, Seguin et al., 2023b). In brain networks, highly connected centrality nodes serve as integrative cores that optimize information flow and orchestrate system-wide coordination, providing global network efficiency and higher-order cognitive functions (Farahani et al., 2019, Sporns, 2018, van den Heuvel and Sporns, 2013, Xu et al., 2022). Notably, alterations in network centrality have been observed at prodromal stages of AD, reflecting early disruptions in brain network organization. Such centrality shifts impair information integration and directly contribute to the characteristic cognitive decline (Dennis and Thompson, 2014, Perovnik et al., 2023). Despite substantial methodological advances in brain network analysis, understanding how information propagates through both direct and indirect signaling pathways remains a significant challenge. In the human connectome, neural communication is not confined to direct anatomical links; instead, signals often traverse multi-step (polysynaptic) routes that collectively shape large-scale functional integration(Park et al., 2021, Seguin et al., 2023a, Seguin et al., 2023b). Traditional centrality metrics—such as degree, betweenness, and eigenvector centrality—have substantially advanced our ability to identify key hubs within core networks such as the default mode network and regions including the cingulate gyrus and precuneus (De Vico Fallani et al., 2014, Kwon et al., 2019, Rubinov and Sporns, 2010). However, these methods primarily quantify local or direct connectivity, making them more sensitive to hubs governed by immediate neighbor interactions, while overlooking globally influential hubs whose importance arises from indirect, long-range signaling. Moreover, secondary hubs embedded in local neural circuits—often critical for maintaining regional integrity—have been less systematically investigated. Consequently, conventional centrality analyses may provide an incomplete characterization of network vulnerability and hierarchical reorganization in neurodegenerative disorders (Binnewijzend et al., 2014, Hojjati et al., 2019). To address these limitations, we introduce DomiRank centrality. This novel metric quantifies the hierarchical dominance of nodes by integrating both direct and indirect interactions, thereby providing a more comprehensive view of network disruption. This novel network metric comprehensively quantifies nodal influence by combining direct and indirect connectivity patterns via iterative signal-propagation modeling (Engsig et al., 2024). We computed DomiRank centrality in the Multicenter Alzheimer’s Disease Imaging (MCADI) cohort (n = 809) and externally validated robustness in an independent Alzheimer’s Disease Neuroimaging Initiative (ADNI) cohort. We then compared DomiRank with seven conventional centralities across two tasks (cross-site disease classification and network robustness analysis). Finally, we integrated the spatial pattern of DomiRank alterations with regional gene-expression profiles to elucidate the molecular basis of these network changes in Alzheimer’s disease (AD).

We hypothesize that DomiRank centrality's unique capacity to capture network-wide nodal influence provides distinct advantages for studying brain functional networks in AD. Specifically, we propose that: (1) DomiRank will highlight a subset of biologically meaningful hubs that partially overlap, but are not identical, to those identified by traditional centrality measures, reflecting its distinct construction; (2) these vulnerable hubs will exhibit significant associations with cognitive decline; and (3) the spatial distribution of DomiRank alterations will correspond to molecular pathways implicated in AD pathogenesis. Collectively, this approach is expected to reveal critical network loci and underlying biological mechanisms that drive AD progression.

## 2 Materials and methods

### 2.1 Participants and image acquisition

This study included resting-state fMRI data from 809 individuals (295 with AD, 257 with Mild Cognitive Impairment (MCI), and 257 healthy controls (HC)), collected across seven centers of the Multicenter Alzheimer's Disease Imaging (MCADI) study. Demographic and clinical characteristics (age, sex, MMSE scores, etc.) are summarized in Table 1. Detailed information is provided in Table S1. Additional information can be found elsewhere in our previous studies (Chen et al., 2023a, Jin et al., 2020, Sun et al., 2024), including inclusion criteria, data quality control, and other modalities such as sMRI and DWI. All procedures adhered to the Declaration of Helsinki and were approved by the relevant institutional review boards (see Method S1). To assess the robustness of our findings, we additionally analyzed data from the Alzheimer's Disease Neuroimaging Initiative (ADNI) cohort (https://adni.loni.usc.edu/), comprising 105 HC and 99 AD participants (demographic and clinical information in Table 2). Among the HC, MCI, and AD groups, there were no statistically significant differences in age or sex ratios. The MMSE score differed significantly among the HC, MCI, and AD groups (*p < 0.001*) (Table 1).

**Table 1:** Demographics and psychological statistics of the included subjects of MCADI.

Site	N	Sex, male/female				Age, mean ± SD				MMSE, mean ± SD			
		HC	MCI	AD	P	HC	MCI	AD	P	HC	MCI	AD	P
S01	119	19/22	13/21	20/24	0.747	68.6 ± 6.7	69.5 ± 8.8	69.9 ± 8.9	0.752	28.5 ± 1.4	26.6 ± 2.5	17.3 ± 6.5	***
S02	69	11/10	11/12	6/18	0.129	68.6 ± 4.7	73.3 ± 8.3	72.8 ± 8.3	0.077	28.9 ± 1.1	27.0 ± 1.8	19.2 ± 4.6	***
S03	94	9/15	10/23	18/19	0.286	65.5 ± 6.2	65.4 ± 8.3	67.7 ± 8.3	0.409	28.8 ± 1.2	25.9 ± 2.5	15.8 ± 5.5	***
S04	126	12/30	8/8	29/37	0.184	65.5 ± 6.8	66.1 ± 7.4	68.0 ± 7.1	0.156	28.5 ± 1.7	24.8 ± 1.5	18.9 ± 3.4	***
S05	208	25/39	46/47	14/25	0.250	66.6 ± 6.3	67.9 ± 10.0	68.8 ± 8.8	0.303	28.1 ± 2.2	24.1 ± 3.7	16.6 ± 6.7	***
S06	73	7/14	10/8	16/17	0.350	65.0 ± 8.2	70.2 ± 7.9	65.5 ± 7.9	0.123	28.5 ± 1.4	21.9 ± 5.0	10.0 ± 6.8	***
S07	120	20/22	13/24	16/25	0.508	68.3 ± 8.0	69.8 ± 6.9	70.2 ± 8.7	0.504	28.8 ± 1.2	26.3 ± 2.6	15.8 ± 5.8	***

***, P < 0.001; χ2-tests tested gender comparisons between groups; one-way ANOVA was applied for age and MMSE score comparisons.

AD, Alzheimer's disease; MCI, Mild Cognitive Impairment; HC, healthy control; MMSE, Mini-Mental State Examination.

**Table 2:** **Information of the replicated ADNI database.** Demographic and neuropsychological data of 105 CE patients and 99 healthy controls of the replication ADNI database.

Site	N	Sex, male/female			Age, mean ± SD			MMSE, mean ± SD		
		HC	AD	P	HC	AD	P	HC	AD	P
2	13	2/7	4/0	0.021	87.8 ± 5.3	73.7 ± 0.4	0.000	28.7 ± 2.6	23.0 ± 1.0	**
6	43	15/11	17/0	0.001	74.8 ± 4.1	77.7 ± 4.4	0.035	29.5 ± 1.0	21.9 ± 2.3	***
13	13	4/5	3/1	0.559	77.4 ± 7.6	76.8 ± 1.1	0.870	29.3 ± 1.1	20.3 ± 1.2	***
18	29	3/18	3/5	0.305	77.7 ± 3.5	73.0 ± 4.3	0.006	28.6 ± 1.3	18.7 ± 5.7	***
19	37	7/7	10/13	0.699	74.1 ± 7.7	79.1 ± 8.4	0.080	28.3 ± 1.9	20.2 ± 3.1	***
31	9	5/0	0/4	0.008	67.9 ± 1.4	56.3 ± 0.4	0.000	29.8 ± 0.5	24.3 ± 2.1	**
53	10	0/5	5/0	0.008	70.0 ± 0.8	71.1 ± 1.4	0.162	28.5 ± 1.3	22.5 ± 4.7	*
130	50	10/0	6/34	0.000	82.4 ± 2.3	73.5 ± 6.9	0.000	29.3 ± 1.0	21.4 ± 4.2	***

***, P < 0.001; **, P < 0.01; *, P < 0.05; χ2-tests tested gender comparisons between groups; one-way ANOVA was applied for age and MMSE score comparisons.

AD, Alzheimer's disease; HC, healthy control; MMSE, Mini-Mental State Examination.

### 2.2 Data preprocessing

**Fig. 1:**
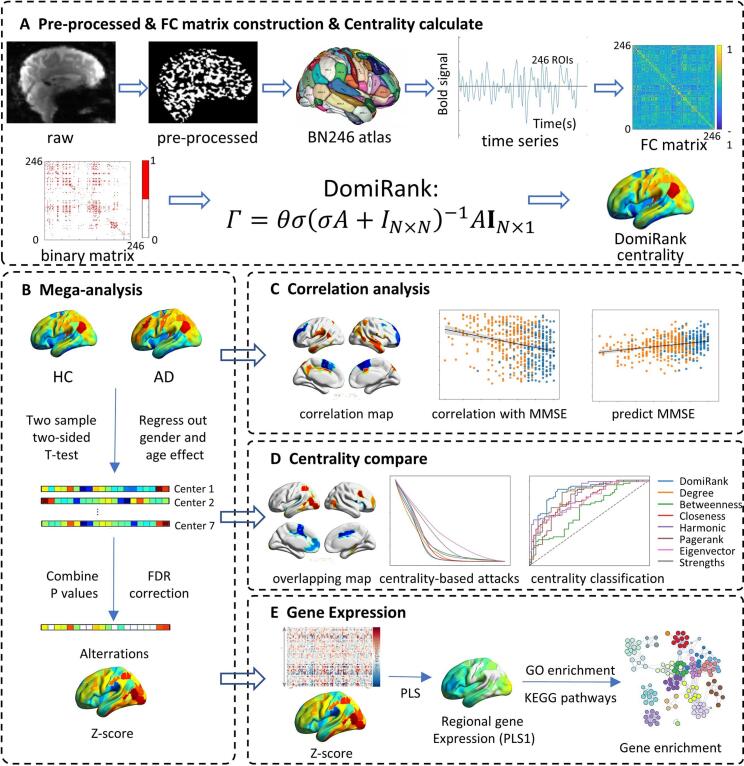
**Pipeline of brain topology alteration analysis.** (A) Calculation of DomiRank centrality: raw rs-fMRI data were preprocessed, parcellated using the Brainnetome atlas, and used to construct functional connectivity matrices for centrality computation. (B) Mega-analysis: group differences between HC and AD were assessed within each center (two-sample *t*-test, controlling for age and gender), and p-values were combined across seven centers with FDR correction. (C) Correlation analysis: associations between DomiRank centrality and MMSE scores, and prediction of diagnostic status and cognitive performance. (D) Centrality comparison: evaluation across regional overlap, network robustness, and classification accuracy against other centrality measures. (E) Gene expression: PLS regression linking centrality alterations to regional gene expression, followed by functional enrichment analysis. AD, Alzheimer's disease; HC, healthy control; MMSE, Mini-Mental State Examination.

Followed our previous studies (Chen et al., 2023a, Jin et al., 2020, Sun et al., 2024), the rs-fMRI scans were pre-processed using the Brainnetome fMRI Toolkit (http://brant.brainnetome.org), which included the following steps: (1) slice-timing correction; (2) realignment to the first volume; (3) normalization (Montreal Neurological Institute [MNI] space with 2 mm × 2 mm × 2 mm); (4) regression of nuisance signals, including linear trends, six motion parameters, and their first-order differences, and signals representing white matter and cerebrospinal fluid and the global mean signal (GSR); Although GSR can induce negative correlations (Liu et al., 2017, Murphy and Fox, 2017), we retained the resulting correlation values and applied proportional thresholding (fixed network density of 4 %) to ensure consistent network sparsity across participants. (5) temporal bandpass filtering (0.01 – 0.08 Hz) to reduce high-frequency noise. Subsequently, any voxel for which the mean absolute deviation in the fMRI signal was less than 0.05 and any area that did not have an fMRI signal recorded from one or more participants was excluded. Additionally, we excluded subjects with significant head motion in any direction exceeding 3 mm or any rotation exceeding 3 degrees. The head motion measures were also included as the concomitant variables. We derived a regional fMRI signal for each region by averaging the fMRI signal across all voxels included in the area parcellated based on the Brainnetome Atlas (Fan et al., 2016). For each participant, we obtained an N × T time series matrix (N = 246 regions of Brainnetome Atlas, T is the time sampling number). The whole workflow is summarized in Fig. 1.

### 2.3 Functional connectivity matrix construction and centrality calculation

The functional connectivity matrix for each subject was obtained by computing Pearson's correlation between the time series of each pair of regions, resulting in a 246 × 246 matrix. To fit a normal distribution, the functional connectivity matrix was transformed using Fisher's z-transformation. The number of edges in the network depends on the network density, which is achieved by thresholding the functional connections between brain regions. To ensure there were no isolated nodes (i.e., brain regions) in the network, the minimum spanning tree method was used to maintain network connectivity. Finally, the correlation matrix threshold was determined (set at 4 % network density; the rationale for this selection is provided in Method S3) and converted to a binary adjacency matrix.

In this study, we employed eight centralities: DomiRank, Betweenness, Closeness, Degree, Eigenvector, Harmonic, PageRank, and Strength. Except for the DomiRank centrality, we used the Python networkx toolkit for the calculations (https://networkx.org/). DomiRank centrality calculation code in Python can be found on GitHub (https://github.com/mengsig/ DomiRank). The specific calculation formula and definition are provided in Method S4.

### 2.4 DomiRank centrality computation

DomiRank centrality $\Gamma \in R^{N}$ is defined as the steady state of the competition–relaxation dynamics $\dot{\Gamma }=\alpha A\left(\theta 1-\Gamma \right)-\beta \Gamma$, which yields the closed‑form $\Gamma =\theta \;\sigma \;{\left(\sigma A+I\right)}^{-1}\;A1$ with σ=α/β>0. For our small adjacency matrix, we compute Γ precisely by solving the linear system (σA+I)Γ=σθA1 rather than using the iterative scheme. In this formulation, θ is a pure scale (fixed to θ = 1 without loss of generality), whereas σ controls the local–global trade‑off and must satisfy 0 <σ < $-1/\lambda_{N}\;$(where $\lambda_{N}$ is the most negative eigenvalue of A). We therefore compute $\lambda_{N}$, scan σ within this feasible interval (e.g., 100 candidates), and either select σ by minimizing the area under the LCC curve for a DomiRank‑based targeted‑attack sequence.

### 2.5 Meta-analysis across different sites

We employed the mega-analysis method to reduce site effects (Eisenhauer, 2020). We first applied two-sample, two-tailed t-tests to compare the two groups at each site. We then used a *meta*-analysis to integrate the multi-center results for each centrality metric. Here, age and gender were controlled using the linear regression model. As suggested in previous studies, the Liptak–Stouffer z-score transform was used to combine p-values across the seven sites, which has optimal power for combining probabilities in mega-analysis (Chen et al., 2023a, Chen et al., 2023b, Jin et al., 2020, Sun et al., 2024). Specifically, the p-values for each data set were transformed into z-scores using the inverse standard normal distribution. In particular, *z_i_ =*φ*^−1^(1 − p_i_ /2)*, where φ*^−1^* is the standard normal cumulative distribution function. The combined z-score was then computed using the Liptak–Stouffer formula (van Zwet and Oosterhoff, 1967):

$$z=\frac{\sum_{i=1}^{k}w_{i}z_{i}}{\sqrt{\sum_{i=1}^{k}w_{i}^{2}}}$$

where w_i_ is the square root of the sample size of dataset i, and k is the number of data sets. Under the null hypothesis, the z-scores follow the standard normal distribution. Therefore, by converting the z-scores to p-values, we identified significant regions that differed between the two groups. The FDR correction was used to control for multiple comparisons across all 246 areas (adjusted significance threshold determined using the Benjamini-Hochberg procedure: p < 0.05 * (246/N), where N is the rank of each region).

### 2.6 Classification and prediction based on centrality measurements

To evaluate whether AD and HC can be distinguished based on centrality metrics, we used a leave-one-site cross-validation framework for each centrality metric, employing the most commonly used Support Vector Machine (SVM) model (Cortes and Vapnik, 1995). Classification performance was evaluated using accuracy (ACC), sensitivity (SEN), specificity (SPE), and the area under the receiver operating characteristic (ROC) curve (AUC). To further validate the classification model's ability to predict clinical indicators, we computed Pearson correlations between the individual pseudoprobabilities of AD risk and MMSE scores in the test set. We also calculated the Pearson correlation between the individual predicted MMSE and actual MMSE values.

### 2.7 Evaluating network robustness under centrality-based attacks

We investigated the effectiveness of different centrality metrics in identifying brain network hubs by simulating attacks that progressively remove nodes in descending order of their centrality. We analyzed how removing these nodes affects network structure and functionality, specifically focusing on the impact on network integrity from removing central nodes. Through this process, we evaluated how well each centrality metric captures the network's most critical nodes. To assess brain network robustness, we used the most common metric, the Largest Connected Component (LCC) (Cats and Krishnakumari, 2020, Trajanovski et al., 2013, Ventresca and Aleman, 2014, Williams and Musolesi, 2016), to assess how the relative size of a network evolved as it underwent sequential node deletions. We directly compared the resulting LCC curves to assess the networks' robustness to different centrality metrics. The measures were then averaged to produce group-level results.

### 2.8 Relationships among clinical scores, gene expression, and centrality

To explore the biological basis of centrality, we performed multiple linear regression between the centrality of each ROI and covariates (MMSE or Montreal Cognitive Assessment (MOCA), age, gender, and site), obtained the T value to evaluate the relationship between cognitive ability and centrality among the covariates (*p* < 0.05 after false discovery rate (FDR) correction).

A gene enrichment analysis was performed in the present study to explore the biological mechanisms underlying the differences in DomiRank centrality between AD and HC. The gene expression data were obtained from the Allen Human Brain Atlas (http://human.brain-map.org/). They were projected to the Brainnetome Atlas using the Abagen toolbox (https://abagen.readthedocs.io/en/stable/), resulting in a 238 × 15,633 gene expression matrix (8 of 246 regions did not identify related genes in the Abagen toolbox). Consistent with the sampling characteristics of the Allen Human Brain Atlas and the default abagen pipeline, left-hemisphere estimates were derived from all six donors. In contrast, right-hemisphere estimates were derived from the two donors with right-hemisphere samples. We first performed partial least squares (PLS) regression to investigate the association between z-scores for AD versus HC based on DomiRank centrality and gene expression. The statistical significance of the variance explained by each PLS component was evaluated using a permutation test with 5000 iterations. To estimate the stability of gene weights, 5000 bootstrap resamples were generated to obtain the distribution of each gene's weights. For subsequent analysis, however, we relied on the gene weights from the full model and ranked all 15,633 genes by their weight magnitudes. Finally, we ranked 15,633 genes according to their corresponding weight value. After that, gene-set enrichment analysis was performed using the top 500 genes with the largest absolute PLS weights, based on the MetaScape platform (Zhou et al., 2019) with FDR correction (*p* < 0.05) (https://metascape.org/gp/index.html#/main/step1). MetaScape integrates more than 40 knowledge bases, including Gene Ontology (GO), KEGG, Reactome, and CORUM, and performs enrichment using a hypergeometric test to identify biological terms that contain significantly more genes than expected by chance. Each input gene is mapped to human-specific annotation resources through Entrez Gene IDs to ensure data consistency. The enriched terms are then clustered based on pairwise Kappa similarities between gene memberships, allowing highly overlapping terms to be grouped into non-redundant clusters. Representative terms from each cluster are visualized as bar plots and enrichment networks, where nodes represent biological processes and edges indicate shared gene content.

## 3 Results

### 3.1 Progressive alterations in DomiRank centrality patterns across HC, MCI, and AD groups

**Fig. 2:**
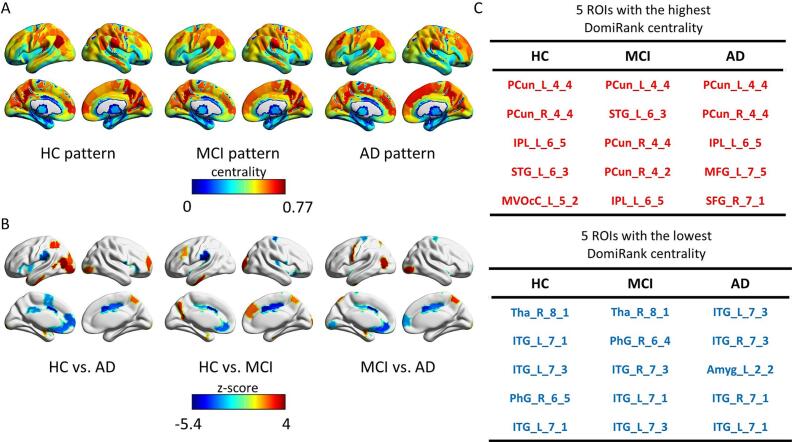
**DomiRank centrality pattern.** (A) DomiRank centrality pattern of HC, MCI and AD. (B) Statistically significant differences in DomiRank centrality between patients with HC versus AD, HC versus MCI, and MCI versus AD. (C) The tables showed the top five large (orange) and low (blue) DomiRank centrality values, along with the associated HC, MCI, and AD groups. (For interpretation of the references to colour in this figure legend, the reader is referred to the web version of this article.)

As shown in Fig. 2A, the HC group exhibited high centrality values predominantly in the angular gyrus, precuneus, and prefrontal cortex. In contrast, regions such as the medial temporal lobe, the occipital cortex, and subcortical areas consistently showed low centrality values. In the MCI group, the overall spatial distribution remained similar to that of the HC; however, the intensity in high-value regions was slightly reduced, while low-value areas became more extensive, particularly in the temporal and occipital regions. In the AD group, the decline became more pronounced, with marked reductions in centrality values in the same high-value areas, alongside a further expansion of low centrality in the medial temporal and posterior cortical regions.

Compared with the HC group, the AD patients showed significant alterations in DomiRank centrality in 29 brain regions (*p_FDR_ < 0.05*) (Fig. 2B). Considerable increases in DomiRank centrality were primarily observed in the left middle and inferior occipital gyri, left superior and inferior parietal gyri, right precuneus, and prefrontal cortex, mainly involving the primary network, particularly the visual network (Vis) and the ventral attention network (VAN). In contrast, significant decreases were detected in the bilateral insula, left supramarginal gyrus, bilateral cingulate gyri, and left supplementary motor area, predominantly within the VAN and the default mode network (DMN). The independent results of the seven centers were consistent with the main results, as shown in Fig. S1.

Compared with the HC group, the MCI subjects exhibited decreased DomiRank centrality in the bilateral cingulate gyri and left supramarginal gyrus. And, the increases in DomiRank centrality were observed in the right superior occipital gyrus, medial paracingulate gyrus, precuneus, as well as the left middle frontal gyrus and precuneus in the MCI group. Compared with the MCI group, the AD patients showed decreased centrality in the bilateral cingulate gyri, precentral gyrus, left fusiform gyrus, and middle frontal gyrus. In contrast, increases were primarily located in the left middle occipital gyrus, right inferior occipital gyrus, and bilateral precuneus.

### 3.2 DomiRank centrality correlates with cognitive scores in AD and MCI

**Fig. 3:**
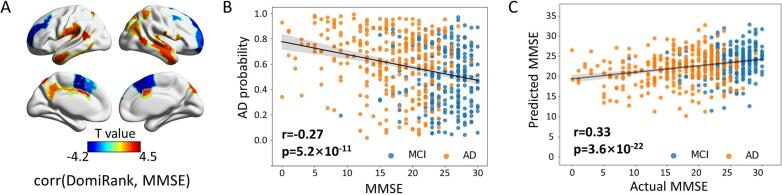
**Classification and individual prediction.** (A) The correlation map between DomiRank centrality and MMSE scores in the AD and MCI patients with FDR correction (p < 0.05). (B) Correlation between predicted AD probabilities and MMSE scores in the test samples. (C) The correlation between predicted and actual MMSE scores of seven centers using leave-one-site-out cross-validation.

After controlling for age, gender, and site, Pearson correlation analyses were performed between the centrality measures and the MMSE in the AD and MCI groups. Twenty-six regions showed significant positive correlations between DomiRank centrality and MMSE scores (*p_FDR_* < 0.05) (Fig. 3A). These regions were located in the precuneus, superior parietal lobe, and temporal lobe. In contrast, DomiRank centrality in 11 regions exhibited significant negative correlations with MMSE scores, mainly involving the medial parietal cortex, including the posterior cingulate cortex and precuneus, as well as parts of the medial frontal cortex. Correlation analysis results between the remaining seven centralities and MMSE scores are presented in Fig. S2. In addition, multiple linear regression revealed 31 regions significantly correlated with DomiRank centrality and MoCA scores (*p_FDR_* < 0.05), with positive correlations primarily located in the bilateral parietal areas (including the superior parietal lobule and supramarginal gyrus) and parts of the frontal lobe, while negative correlations were observed in the medial parietal and posterior cingulate, a distribution pattern similar to that of MMSE (Fig. S3).

### 3.3 DomiRank centrality predicts diagnostic status and cognitive scores

Using a leave-one-site-out cross-validation approach, we trained an SVM model to predict diagnostic status and clinical MMSE scores from centrality metrics for individuals. Importantly, we found a significant negative correlation between individual pseudoprobabilities and cognitive performance in AD and MCI subjects (r = −0.27, p < 0.001 for both AD and MCI, and r = −0.20, *p < 0.001* for AD). For disease progression, the average correlation coefficient between the predicted and actual MMSE scores, RMSE (root mean squared error), and R^2^ (explained variance) across the seven test sets was 0.33 (*p* < 0.001), 6.25, and 0.15, respectively.

### 3.4 DomiRank centrality effectively identifies network alterations associated with Alzheimer's disease

**Fig. 4:**
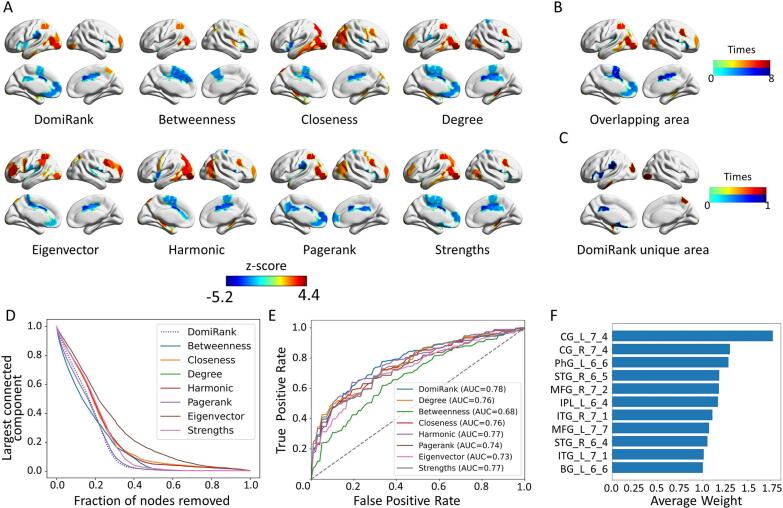
**Centrality comparison.** (A) Statistically significant differences in 8 different centralities between patients with HC versus AD. (B) Overlapping area of significant difference in the eight centralities (reserved only for more than 4, where the negative significance difference is reserved as negative). (C) Unique regions of DomiRank centrality compared with overlapping regions. (D) Centrality-based attacks on brain networks. (E) ROC and AUC of the 8 centrality measures based on leave-one-site-out classification. (F) The largest feature region weights in the classification task based on DomiRank centrality.

A *meta*-analysis was performed to test differences in eight centralities between AD and HC. Fig. 4A shows the regions associated with significant differences in eight centralities between the AD and HC groups (*p_FDR_ < 0.05*). Regions exhibiting significantly increased centrality in AD were predominantly located in the left occipital, temporal, and parietal lobes, whereas significantly decreased centrality was primarily observed in the cingulate gyrus, as well as the frontal and parietal lobes. In addition, independent analyses were performed within each of the seven centers (Fig. S4), and the results demonstrated high consistency across centers. Regions with significantly reduced DomiRank centrality affected by AD were primarily located in the bilateral parietal and frontal cortices, as well as parts of the cingulate gyrus.

Next, the overlapping areas of the eight centralities and the unique regions identified by DomiRank centrality were further analyzed. The areas with decreased centrality were concentrated in the frontal lobe and the cingulate gyrus (Fig. 4B), regions among the most affected by AD. As shown in Fig. 4C, DomiRank uniquely detected reductions primarily within the frontoparietal network (FPN), parietal lobe, insula, and basal ganglia.

In terms of network robustness evolution, the network damage rate based on DomiRank ranked among the top three and even took first place in the 20 %-35 % stage (Fig. 4). As shown in Fig. 4E, the eight centralities were used for the AD classification by the leave-one-site-out cross-validation method, and the AUC results showed that the DomiRank centrality had the best performance (average AUC = 0.78, ACC = 0.69, SEN = 0.73, SPE = 0.66) in randomized cross-validation. In addition, under different thresholds, DomiRank centrality achieved similar results in network robustness evolution and classification tasks (Figs. S4-S6). We further assessed and ranked the importance of individual brain regions in classification and prediction. Notably, the most essential nodes are located in the frontal and temporal lobes, as well as subcortical structures such as the left cingulate gyrus and the striatum within the basal ganglia (Fig. 4).

### 3.5 DomiRank centrality highlights synaptic signaling–related genetic signatures in AD

**Fig. 5:**
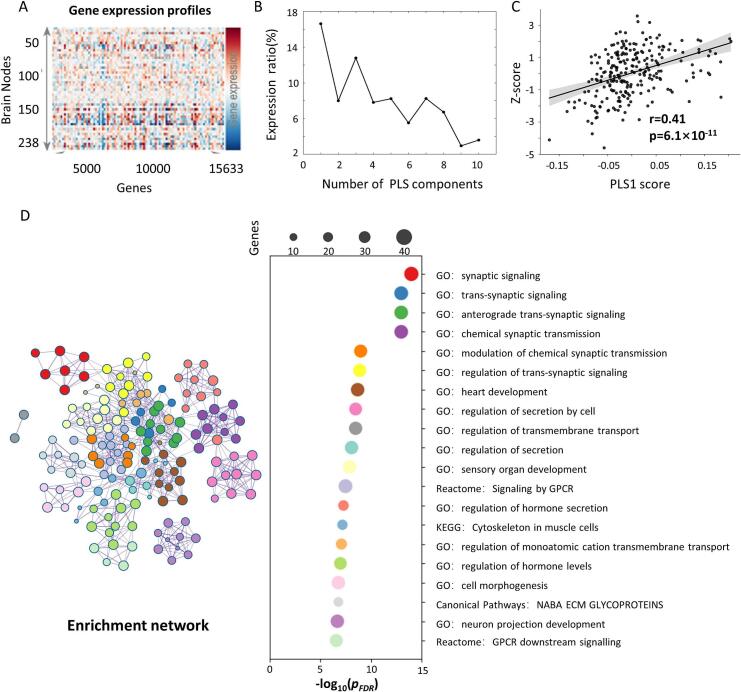
**Association between DomiRank centrality differences and gene expression profiles.** (A) Gene expression profiles across brain nodes. (B) Explained ratios for the first 10 components obtained from the PLS regression analysis. (C) Spatial association between case–control differences in modular variability and PLS1 scores. (D) Gene enrichment network for PLS1 genes.

A significant spatial association was found between AD-related changes, as determined by DomiRank centrality, and nodal gene expression profiles (Fig. 5A). The first PLS component (PLS1) accounted for 16.6 % of the variance in gene expression, and this effect was statistically significant (permutation test, p < 0.001). It showed significant correlation with the T-map of DomiRank centrality in HC versus AD (r = 0.41, p = 6.1e-11) (Fig. 5).

**Fig. 6:**
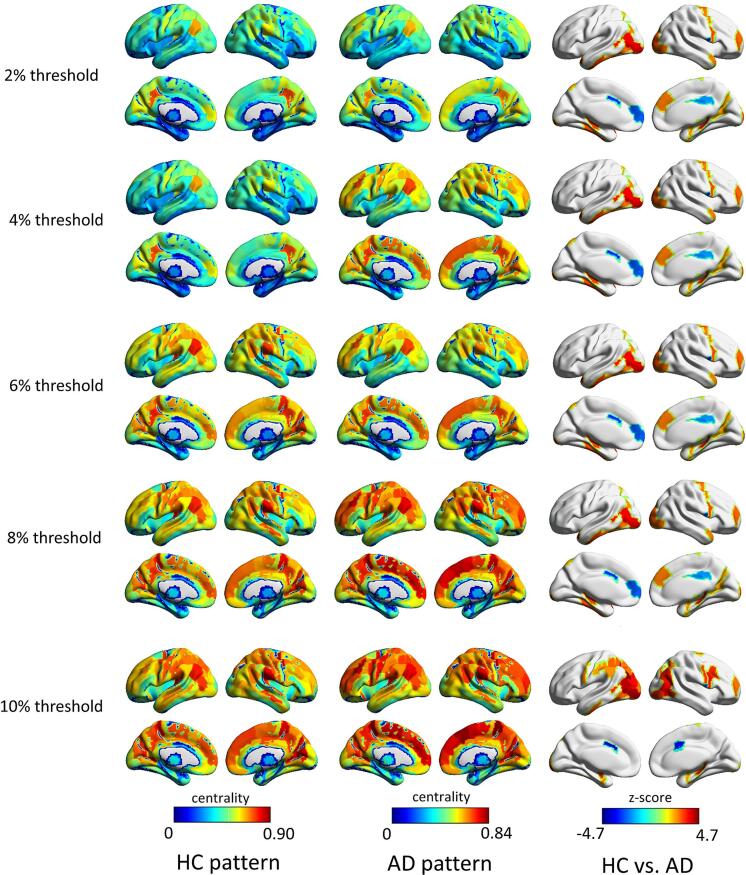
**The effect of different thresholds on the main result.** The similarities between the HC patterns at the 2 %, 6 %, 8 %, and 10 % thresholds and the HC pattern at the 4 % threshold were 0.98, 0.99, 0.98, and 0.97, respectively. Comparable values were observed for the AD patterns, with similarities of 0.98, 0.99, 0.98, and 0.97. For the z-score maps reflecting HC–AD differences, the corresponding similarities with the 4 % threshold result were 0.84, 0.91, 0.88, and 0.84.

We found that synaptic signaling (GO:0099536, *p_FDR_* = 1.15e-14), *trans*-synaptic signaling (GO:0099537, *p_FDR_* = 5.01e-14), anterograde *trans*-synaptic signaling (GO:0098916, *p_FDR_* = 8.99e-14), and chemical synaptic transmission (GO:0007268, *p_FDR_* = 8.99e-14) (Fig. 6D) were the most significantly correlated biological processes with the DomiRank centrality differences (Table S7).

### 3.6 DomiRank centrality shows consistent results across thresholds, datasets, and atlases

To examine the reproducibility of different thresholds, network analyses were conducted using sparsity thresholds of 2 %, 6 %, 8 %, and 10 % (Fig. 6). The spatial distributions of HC and AD patterns were highly consistent with those obtained at the 4 % threshold, with the highest centrality regions mainly located in the prefrontal, parietal, and occipital association cortices, and correlation coefficients exceeding 0.95. In addition, the z-score maps representing HC–AD differences also showed high consistency, with significantly decreased centrality primarily observed in the cingulate cortex and medial temporal regions, and correlation coefficients ranging from 0.84 to 0.90. These findings indicate that the main results are stable across a range of sparsity thresholds.

In addition, we validated the reproducibility of DomiRank centrality using a public ADNI dataset. The HC pattern showed high centrality values primarily in the prefrontal, parietal, and occipital association cortices, with a similarity of 0.83 to the main results (Fig. S7). The AD pattern showed a distribution of high centrality regions comparable to that in the main results, mainly in the frontal and parietal cortices, with a similarity of 0.81. Group comparisons revealed significantly decreased centrality in the cingulate cortex, temporoparietal junction, and medial temporal regions, which was consistent with the main findings. These results further demonstrated the cross-dataset robustness of DomiRank centrality.

When using different brain atlases, the similarity of the difference maps with the main results was r = 0.50 for the Willard 470 atlas and r = 0.57 for the Schaefer 1000 atlas (cortical regions only) (Fig. S8). Under the absolute threshold approach, the difference map exhibited a nearly identical spatial distribution to the main results, with a correlation coefficient of r = 0.99 (Fig. S9). Regarding the length of the time series, the difference map derived from the first 170 frames remained highly consistent with the main results, with a similarity of r = 0.92 (Fig. S10). Collectively, these findings confirm that the HC pattern, AD pattern, and their difference maps are robust across thresholding strategies, atlas choices, and time series lengths, underscoring the exceptional reproducibility of DomiRank centrality.

## 4 Discussion

In this study, we introduced DomiRank centrality to explore brain topology alteration in the brain networks of AD. We applied this innovative centrality to analyze a large-scale, multicenter rs-fMRI dataset comprising AD patients and matched HCs to identify hub disruptions associated with the disease. By comparing DomiRank centrality with conventional centralities, we demonstrated its sensitivity for detecting AD-related network alterations. Furthermore, we evaluated the clinical utility of DomiRank centrality using machine learning classification models, which achieved higher accuracy in distinguishing AD patients from HCs than models based on traditional centrality metrics. DomiRank not only identified canonical hub regions typically captured by traditional centrality metrics but also pinpointed functionally pivotal regions—such as the basal ganglia—that served as integrative hubs and were often notably affected in AD. Correlation analysis between DomiRank centrality and standardized cognitive assessments revealed significant associations with clinical severity. Gene enrichment analysis of DomiRank-identified hubs showed enrichment for AD-relevant biological pathways. Collectively, DomiRank centrality is a valuable new tool for characterizing network dysfunction in AD, potentially advancing both our theoretical understanding of the disorder's pathophysiology and practical applications in diagnosis and prognosis.

### 4.1 DomiRank centrality aligns with traditional centrality metrics in capturing core AD-related brain network hubs

This study found that both DomiRank centrality and traditional centrality can accurately identify hubs in brain networks. Based on DomiRank centrality, the spatial distribution of the top-ranked hubs (mainly located in the frontal and temporal cortices, including the angular gyrus and supramarginal gyrus) remained relatively stable in the HC, MCI, and AD groups, but their corresponding centrality values decreased significantly with disease progression. In contrast, regions with consistently low centrality were primarily located in the medial temporal lobe, including the inferior temporal gyrus and the parahippocampal gyrus, as well as subcortical structures such as the thalamus and the amygdala. In the MCI group, these low-value areas are evident, with the thalamus and medial temporal regions being most affected. In the AD group, low centrality became more concentrated in the inferior temporal gyrus and extended into the amygdala, highlighting the progressive involvement of medial temporal and limbic regions as the disease advances. Notably, this was observed under a controlled condition where the number of network connections (threshold) was kept constant across subjects. This finding suggested that in AD, hub regions do not undergo structural displacement, but rather lose their functional dominance within the brain network (Guillon et al., 2017, Guillon et al., 2019, Hu et al., 2022, Suhui et al., 2024, Taguas et al., 2024). Furthermore, intergroup comparisons revealed that the precuneus and temporal lobes consistently exhibited significant reductions in DomiRank centrality across all three pairwise comparisons. These areas were known to be key components of the default mode network (DMN)—a system frequently reported to be functionally impaired in AD. DomiRank centrality evaluates the importance of nodes based on the global network structure, making it highly sensitive to the identification of brain transit hubs (Lanciego et al., 2012, Middleton and Strick, 2000). The consistent involvement of these regions underscores DomiRank's sensitivity to vulnerable and functionally declining hubs, making it a powerful tool for characterizing disease-related network disruptions (Binnewijzend et al., 2014, Jin et al., 2020, Kang et al., 2019, Ries et al., 2008).

### 4.2 DomiRank uniquely identifies AD-associated local hubs overlooked by conventional centrality metrics

DomiRank centrality was initially developed with the primary aim of enabling efficient targeted attacks to dismantle network structures and disrupt their function (Engsig et al., 2024). DomiRank integrated local and global topological information via adjustable parameters, which were more sensitive to network disruption than traditional centralities that considered only first-order nodes. This meant that DomiRank centrality could not only accurately identify traditional network hubs but also local hubs and hubs in link loops (Engsig et al., 2024). Based on DomiRank centrality, the areas most significantly affected by AD included not only the traditional core areas but also information relay centers, specifically the basal ganglia. The basal ganglia were subcortical regions that served as relay centers, integrating primary signals and transmitting them to higher functional areas (mainly the frontal lobe). DomiRank identified unique regions with significant differences, including the striatum and globus pallidus. The striatum was the key entrance of the cortex-striatum-thalamus-cortex loop and was involved in motor planning, reward perception, habit formation, and cognitive regulation. The globus pallidus, as an output structure, was responsible for transmitting information out of the basal ganglia and regulating the activities of the thalamus and the cerebral cortex (Lanciego et al., 2012, López-Ojeda and Hurley, 2025, Middleton and Strick, 2000, Provost et al., 2015).

### 4.3 DomiRank-derived network anomalies closely track the progression trajectory of AD

To further demonstrate the advantages of DomiRank centrality, we evaluated its performance in both disease classification tasks and network robustness analysis. Among various centrality measures, DomiRank achieved the highest performance in binary classification (Fig. 4E). This was attributed to its excellent ability to rank nodes by importance, particularly in assigning accurate centrality values to medium- and high-importance nodes, such as network hubs or information relay centers, which were usually key contributors to classification. This was evident in the classification importance sorting (Fig. 4F), where high-importance nodes came not only from the network hubs but also from other relay information centers (Bostan and Strick, 2018, Jackson and Bernard, 2023, Lanciego et al., 2012). In addition, in the network robustness destruction (Fig. 4D), DomiRank centrality was in the optimal range for the first 20 % and then reached the first position. This was because DomiRank centrality provided a more accurate positioning of the less critical nodes, namely, information relay centers.

### 4.4 AD-relevant synaptic signaling genetic signatures underpin domiRank-revealed network disruptions

Based on the gene expression characteristics of vulnerable brain regions identified by DomiRank centrality, gene enrichment analysis (GEA) revealed multiple biological processes closely related to the core pathological mechanisms of AD. The most significantly enriched pathway was synaptic signaling, with subcategories including transsynaptic signaling, chemical synaptic transmission, and anterograde transsynaptic signaling. Synaptic signaling was crucial in learning and memory. These functional abnormalities may have been related to the obstruction of Aβ (β-amyloid protein) and Tau protein propagation and abnormal aggregation in AD (Chételat et al., 2020, Nguyen et al., 2024, Pignataro and Middei, 2017, van der Kant et al., 2019, Vogel et al., 2020). In addition, differentially expressed genes were enriched in processes such as regulation of cell secretion and regulation of transmembrane transport, suggesting that these brain regions might be involved in the release of inflammatory factors, neurotransmitter metabolism, and Aβ transmembrane clearance (Hansen et al., 2018, Heneka et al., 2015). Abnormalities in transmembrane transport might have affected intracellular and extracellular calcium ion homeostasis and energy metabolism, while reducing the clearance of Aβ from the brain (Buechler and Salinas, 2018, Demuro et al., 2005, Popugaeva et al., 2017, Supnet and Bezprozvanny, 2010). Understanding these potential mechanisms is crucial to developing therapeutic strategies to slow AD progression.

## 5 Limitations

First, the HC/MCI/AD classifications in both the MCAD and ADNI datasets were determined based on clinical evaluations and neuropsychological assessments conducted by experienced clinicians. However, neither dataset systematically incorporated cerebrospinal fluid (CSF) or positron emission tomography (PET) biomarkers for diagnostic confirmation. Although a subset of ADNI participants has available Aβ and tau biomarker measurements, these data were not used as diagnostic criteria. As a result, a certain degree of diagnostic misclassification cannot be fully excluded. Second, the classification accuracy of DomiRank centrality varied significantly across centers; further validation in larger, more diverse cohorts was needed to assess its robustness. Thirdly, the lack of longitudinal data prevented us from directly examining how DomiRank centrality scores evolved and their relationship to disease progression. The inclusion of longitudinal observations would help clarify whether DomiRank centrality could capture meaningful temporal dynamics in neurodegenerative diseases. Finally, the AHBA gene expression data were obtained from an independent cohort rather than the individuals who provided the MRI data. This approach assumes that the spatial distribution of gene expression is consistent across individuals. Therefore, while our findings offer meaningful insights into the spatial correspondence between brain organization and gene expression, caution should be taken when generalizing these results to individual-level inferences or other populations.

## 6 Conclusion

In this study, we utilized DomiRank centrality to investigate the alterations in brain topology within the AD brain network. This new centrality evaluation successfully integrated both global and local information, providing a theoretical basis for accurately evaluating brain network hubs. Compared with other centralities, DomiRank centrality not only identified the main central areas similar to traditional centrality, but also identified hub areas such as the basal ganglia and cingulate gyrus, which were highly correlated with the results of gene enrichment analysis. Collectively, these findings suggest that DomiRank centrality is a valuable new tool for characterizing network dysfunction in AD, potentially advancing both our theoretical understanding of the disorder's pathophysiology and practical applications in diagnosis and prognosis.

## 7 Code availability

In the current study, the fMRI preprocessing software BRANT has been open-sourced on YongLiuLab (https://github.com/YongLiuLab), and all other computational toolkits and code have been cited in the Methods section.

## CRediT authorship contribution statement

**Longhao Ma:** Writing – original draft, Visualization, Software, Methodology, Formal analysis, Data curation. **Pan Wang:** Writing – original draft, Visualization, Validation, Resources, Methodology, Investigation, Data curation. **Dawei Wang:** Resources, Project administration. **Hongxiang Yao:** Resources, Project administration, Data curation. **Bo Zhou:** Resources, Project administration, Data curation. **Yonghua Zhao:** Funding acquisition, Project administration. **Zhengluan Liao:** Resources, Project administration, Data curation. **Yan Chen:** Resources, Project administration, Data curation. **Xi Zhang:** Resources, Project administration, Data curation. **Ying Han:** Resources, Project administration, Data curation. **Jie Lu:** Resources, Project administration, Data curation. **Kun Zhao:** Writing – review & editing, Validation, Funding acquisition. **Yihe Zhang:** Writing – review & editing, Writing – original draft, Validation, Supervision, Methodology, Funding acquisition. **Yong Liu:** Writing – review & editing, Validation, Supervision, Project administration, Methodology, Funding acquisition.

## Funding

This work was partially supported by the 10.13039/501100001809National Natural Science Foundation of China, China (Nos. 62333002 and 82172018), the Beijing Municipal Natural Science Foundation, China (No. 7244519), the 10.13039/501100005090Beijing Nova Program, China (20250484966), the Science and Technology Development Fund, Macau SAR, China (No. 0065/2023/RIB3), the 10.13039/501100012226Fundamental Research Funds for the Central Universities, China (2024RC11).

## Declaration of competing interest

The authors declare that they have no known competing financial interests or personal relationships that could have appeared to influence the work reported in this paper.

## Data Availability

All data mentioned in this study were acquired conditionally from the corresponding author.
