# Supplementary material for: Brain topology alteration in Alzheimer’s disease brain networks: A multi-center study
Source: Neuroimage Clin. 2025 Nov 30;49:103919. doi: 10.1016/j.nicl.2025.103919 (PMC12753252; doi:10.1016/j.nicl.2025.103919)
Supplement: Supplementary Data 1 [file mmc1.docx]

Supplementary Materials for

**Brain topology alteration in Alzheimer's disease brain networks: A multi-center study**

**Outline**

[Supplementary Methods 2](#_Toc15719)

[Method S1. Subjects Information 2](#_Toc22710)

[Method S2. Image acquisition 7](#_Toc17881)

[Method S3. Threshold selection 8](#_Toc23727)

[Method S4. Centrality formula and introduction. 9](#_Toc27423)

[Supplemental Tables 11](#_Toc15852)

[Table S1. fMRI scanner and image-acquisition protocol information for MCAD 11](#_Toc9821)

[Table S2. The statistical results of the classification 12](#_Toc30959)

[Table S3. Detailed statistical results of gene expression 13](#_Toc4664)

[Supplemental Figures 15](#_Toc13647)

[Figure S1. Group differences for each center. 15](#_Toc22887)

[Figure S2. Centralities difference and correlation of MMSE. 17](#_Toc30326)

[Figure S3. Correlation between DomiRank centrality and MoCa. 18](#_Toc12021)

[Figure S4. Centrality-based attacks based on different threshold. 19](#_Toc4969)

[Figure S5. Classification task based on DomiRank centrality at different thresholds. 20](#_Toc17771)

[Figure S6. Classification task based on eight centralities at different thresholds. 21](#_Toc13754)

[Figure S7. Main results of different datasets. 22](#_Toc9341)

[Figure S8. The effect of different brain atlases on the main result. 23](#_Toc24848)

[Figure S9. The effect of the absolute threshold on the main result. 24](#_Toc30962)

[Figure S10. The effect of the length of the time series on the main result. 25](#_Toc20459)

[References 26](#_Toc6356)

# Supplementary Methods

## Method S1. Subjects Information

The Multi-Center Alzheimer's Disease Imaging Consortium Dataset (MCADI) is a multi-center cohort comprising data from seven sites (AD: *n* = 295, MCI: *n* = 257, NC: *n* = 257). All the subjects underwent general physical, psychological, and laboratory examinations before enrollment in the formal study. The subjects took the Mini-Mental State Examination (MMSE). This data usage extended our previous studies and included a new site from the PLA hospital with the same protocols. Detailed information, including ethics and the inclusion criteria, can be found elsewhere in our previous studies and the supplemental materials ([Jin et al., 2020](#_ENREF_12); [Li et al., 2019](#_ENREF_14); [Qu et al., 2021](#_ENREF_25); [Shi et al., 2023](#_ENREF_26)).

The data introduction can be found in the supplemental material of previous papers ([Chen et al., 2023](#_ENREF_1); [Jin et al., 2020](#_ENREF_12); [Sun et al., 2024](#_ENREF_28)). Here, with permission, we have rewritten the information to maintain the integrity of the present study.

### PL_S1, PL_S2 and PL_G (S01, S02, S07)

This study was approved by the Medical Ethics Committee of PLA General Hospital. Written informed consent was obtained from each enrolled subject or his/her authorized guardian. All of the participants were recruited by an advertisement (<http://www.301ad.com.cn>, Chinese version). Before selection for this study, all of the participants were given free physical, psychological, and laboratory examinations. All patients received professional suggestions for further treatment.

All of the subjects were right-handed and underwent a battery of neuropsychological tests, including the Mini-Mental State Examination (MMSE) ([Folstein et al., 1975](#_ENREF_5)), auditory verbal learning test (AVLT), Geriatric Depression Scale (GDS) ([Yesavage et al., 1982](#_ENREF_34)), Clinical Dementia Rating (CDR) ([Morris, 1993](#_ENREF_21)), and Activities of Daily Living (ADL) Scale ([Lawton and Brody, 1969](#_ENREF_13)). In brief, the AVLT consisted of 1 learning trial in which a list of 10 Chinese double-character words were read and the subject was asked to immediately recall as many items as possible. The trial was repeated twice, and the immediate recall score was the average of 3 accurate recalls. After a 5-minute delay, each subject was asked to recall the words from the initial list (AVLT-delayed recall). The subjects were then told to identify the 10 studied words, which were inter-mixed with 10 novel words (AVLT-recognition).

The recruited AD patients fulfilled the following inclusion criteria: (1) diagnosed using the National Institute of Neurological and Communicative Disorders and Stroke and the Alzheimer Disease and Related Disorders Association criteria for probable AD; (2) CDR = 1 or 2; (3) currently receiving no tropic drugs, such as cholinesterase inhibitors; and (4) able to perform the neuropsychological tests and tolerate MR scanning.

The diagnostic criteria for MCI were determined as previously described([Petersen et al., 1999](#_ENREF_24)) and included the following: (1) memory complaints lasting at least 6 months; (2) CDR = 0.5; (3) intact functional status and ADL< 26; and (4) lack of dementia. The criteria for NC included the following: (1) normal physical status; (2) CDR = 0; and (3) without memory complaints.

The following exclusion criteria were used in this study: (1) metabolic conditions such as hypothyroidism or vitamin B12/folic acid deficiencies; (2) psychiatric disorders such as schizophrenia or depression; (3) infarction or brain hemorrhaging, as indicated by MR/CT imaging; and (4) Parkinsonian syndrome, epilepsy, or other nervous system diseases that can influence cognitive function. Additionally, patients with a metallic foreign body, such as a cochlear implant or heart stent, or those with other relevant contraindications for MR scanning, were excluded from the study.

Related publications can be found elsewhere ([Feng et al., 2018](#_ENREF_4); [Guo et al., 2014](#_ENREF_8); [Wang et al., 2015](#_ENREF_30); [Yao et al., 2014](#_ENREF_33); [Zhang et al., 2014](#_ENREF_35); [Zhou et al., 2015](#_ENREF_36)).

### HH_Z (S03)

The dataset followed the same protocol as PL_G and PL_S. This study was approved by the Medical Ethics Committee of Tianjin Huanhu Hospital. The patients were recruited from the memory clinic of the Neurology Department of Tianjin Huanhu Hospital, Tianjin, China. The control subjects were recruited from the local community using advertisements. Written informed consent was obtained from each enrolled subject or his/her authorized guardian. The participants underwent general physical, psychological, and laboratory examinations prior to enrollment in the formal study. The participants did not undergo the auditory verbal learning test. The participants did not take medications that might have influenced cognition during the scans, and all patients received professional suggestions for further treatment.

### QL_W (S04)

The dataset followed the same protocol as PL_G and PL_S. This study was approved by the Medical Ethics Committee of Qilu Hospital of Shandong University. The patients were recruited from the memory clinic of the Department of Neurology and Radiology, Qilu Hospital of Shandong University, Ji’nan, China. The control subjects were recruited from the local community using advertisements. Written informed consent was obtained from each enrolled subject or his/her authorized guardian. The participants underwent general physical, psychological, and laboratory examinations prior to enrollment in the formal study. The participants did not take medications that might have influenced cognition during the scans, and all patients received professional suggestions for further treatment.

### XW_H (S05)

The study was approved by the Medical Research Ethics Committee and Institutional Review Board of Xuanwu Hospital (ClinicalTrials.gov identifiers: NCT02353884 and NCT02225964). Part of the data has been used in several previous studies, and detailed information can be found elsewhere ([Li et al., 2014](#_ENREF_15); [Yan et al., 2018](#_ENREF_32)).

All subjects underwent a series of standardized clinical evaluations, including a medical history interview, neurologic examination, and a battery of neuropsychological tests. The neuropsychological tests included the Chinese version of the MMSE, the Beijing version of MoCA ([Lu et al., 2011](#_ENREF_18)), the CDR ([Morris, 1993](#_ENREF_21)), the AVLT ([Guo et al., 2007](#_ENREF_7)), an ADL assessment([Lawton and Brody, 1969](#_ENREF_13)), the Hachinski Ischemic Scale ([Hachinski et al., 1975](#_ENREF_9)), the Hamilton Depression Rating Scale (HAMD) ([Hamilton, 1960](#_ENREF_10)), and The Center for Epidemiologic Studies Depression Scale ([Dozeman et al., 2011](#_ENREF_3)). Confirmation of diagnosis for all subjects was made by the consensus of at least two experienced neurologists in the Neurology Department of Xuanwu Hospital. The diagnoses were based on the available data from the neuropsychological assessment evaluation, a battery of general neurological examinations, and subject symptoms as well as functional capacity reports.

The inclusion criteria for aMCI diagnosis included the following ([Petersen, 2004](#_ENREF_22)): (a) memory complaints, confirmed by an informant; (b) objectively impaired memory confirmed by neuropsychological tests; (c) a definite history of cognitive decline; (d) not meeting the criteria for dementia according to the Diagnostic and Statistical Manual of Mental Disorders, Fourth Edition, Revised (DSM-IV-R); and (e) a CDR score of 0.5.

AD subjects were diagnosed according to the National Institute of Aging-Alzheimer's Association (NIA-AA) criteria for clinically probable AD ([McKhann et al., 1984](#_ENREF_19); [McKhann et al., 2011](#_ENREF_20)): (a) meeting the criteria for dementia; (b) insidious and gradual onset (not sudden) over more than 6 months; (c) definite history of declining cognition; (d) initial and most prominent cognitive deficits evident in amnestic or non-amnestic performance; and (e) hippocampal atrophy confirmed by structural MRI.

### XW_Z (S06)

The NC patients were required to meet the following research criteria: (a) no memory concerns; (b) MMSE and MoCA scores within the normal range (adjusted for age, sex, and education); and (c) a CDR score of 0.

The exclusion criteria applied to all subjects included the following: (a) vascular cognitive impairment (Hachinski Ischemic Scale score > 4 points); (b) severe depression (HAMD score > 24 points or The Center for Epidemiological Studies Depression Scale score > 21 points); (c) other central nervous system diseases that could cause cognitive decline (e.g., epilepsy, brain tumors, Parkinson's disease, or encephalitis); (d) systemic diseases that could cause cognitive impairments (e.g., anthracemia, syphilis, thyroid dysfunctions, severe anemia, or HIV); (e) a history of psychosis or congenital mental growth retardation; (f) severe hypopsia or dysacusis; (g) cognitive decline caused by traumatic brain injury; (h) severe end-stage disease or severe diseases in acute stages; (i) a history of stroke; or (j) inability to complete neuropsychological tests or with a contraindication for MRI.

All the participants were recruited by advertisement and supported throughout the testing procedures in a specialist neuropsychological research facility at Xuanwu Hospital, Beijing, China. Patients and informants (usually family members) were interviewed clinically by a senior psychiatrist (X. Zhang). Written consent forms were obtained from all subjects or their legal guardians (usually a family member). The study was approved by the Ethics Committee of Xuanwu Hospital. AD subjects were diagnosed using standard operationalized criteria (DSM-IVR [American Psychiatric Association, 1994] and NINCDS-ADRDA ([McKhann et al., 1984](#_ENREF_19))).

The inclusion criteria for the AD diagnosis included the following: severity of dementia was assessed using the Clinical Dementia Rating (CDR) scale([Morris, 1993](#_ENREF_21)). Patients with a diagnosis of AD and CDR score of 1 were classified as mild AD; patients with a CDR score of 2 or 3 were diagnosed as severe AD.

Mild cognitive impairment (MCI) was diagnosed according to standard criteria ([Choo et al., 2007](#_ENREF_2); [Petersen et al., 2001](#_ENREF_23); [Petersen et al., 1999](#_ENREF_24)), which included subjective memory loss with objective evidence of memory impairment in the context of normal or near-normal performance on other domains of cognitive functioning; minimal impairment of activities of daily living; and a CDR score of 0.5. Normal volunteers had a CDR score of 0.

All participants satisfied the following inclusion criteria: (1) no history of an affective disorder within one month before assessment; (2) normal vision and audition; (3) able to cooperate with cognitive testing; (4) aged between 50 and 90 years; (5) no clinical history of stroke or other severe cerebrovascular diseases; and (6) no more than one lacunar infarction, without patchy or diffuse leukoaraiosis, on neuroradiological assessment of conventional MR images.

The exclusion criteria included the following: (1) severe general medical disorders of cardiovascular, endocrine, renal, or hepatic systems; neurological disorders associated with potential cognitive dysfunction, including local brain lesions, traumatic brain injury with loss of consciousness or confusion, and dementia associated with neurosyphilis, Parkinsonism, or Lewy body disease; psychiatric disorders including depression, alcohol, or drug abuse; (2) concomitant use of psychotropic medication in a large quantity; and (3) insufficient cognitive capacity to understand and cooperate with study procedures.

All patients underwent a complete physical and neurological examination, an extensive battery of neuropsychological assessments, and standard laboratory tests. Healthy volunteers underwent a brief clinical interview and MMSE to confirm that they satisfied the exclusion criteria for cognitive deficits, psychoactive drug use, and clinical disorders.

This detailed information can be found elsewhere in our previous studies ([He et al., 2014](#_ENREF_11); [Liu et al., 2016](#_ENREF_16); [Liu et al., 2014](#_ENREF_17); [Song et al., 2013](#_ENREF_27); [Wu et al., 2016](#_ENREF_31)).

## Method S2. Image acquisition

In the MCADI, participants were scanned with MRI scanners at seven sites. The data obtained 3D T1-weighted images, resting-state functional images, and Diffusion Tensor Imaging (DTI) ([Qu et al., 2021](#_ENREF_25); [Sun et al., 2024](#_ENREF_28)). In this study, we only used fMRI data. The corresponding MRI acquisition protocols are described in Table S1.

To minimize the variation in head motion, we excluded the subjects with large head motion in any direction corresponding to >3 mm or any rotation >3°.

In the ADNI, participants were acquired using Philips Medical Systems Scanners and they underwent control at the Mayo Clinic. The fMRI images were obtained with a field strength of 3.0 Tesla, a repetition time of 3 s, an echo time of 30 ms, a flip angle of 80◦, matrix 64 × 64, 140 volumes,48 slices per volume, and a slice thickness of 3.3 mm. The voxel size was 3.3 × 3.3 × 3.3 mm3. For further details on MRI acquisitions, refer to the “MRI scanner protocol” at ADNI site.

## Method S3. Threshold selection

To convert a functional connectivity matrix into a binarized connectivity matrix, we needed to select a threshold to determine effective connections. Therefore, threshold selection was critical: setting it too low could prevent the centrality metric from identifying certain local hubs due to an insufficient number of effective connections, whereas setting it too high could lead to many unimportant nodes being misclassified as hubs. Recent work shows that global efficiency varies systematically with threshold and exhibits an "elbow" at around ~4%; accordingly, we treat 4% as the primary density and verify stability across a range of sparsity levels rather than relying on a single cut-off ([Garrison et al., 2015](#_ENREF_6); [van den Heuvel et al., 2017](#_ENREF_29)). To construct a sparse functional connectivity network, we set the sparsity threshold range from 2% to 10% with 2% increments. For each sparsity level, Pearson correlation coefficients were ranked in descending order, and the top-ranked connections corresponding to the specified sparsity were retained and assigned aij=1, while the remaining connections were set to aij=0.

## Method S4. Centrality formula and introduction.

| Centrality | Formula | Description | Interpretation |
| --- | --- | --- | --- |
| Betweenness | $C_{B}\left( v \right)=\sum_{s\neq v\neq t} \frac{\sigma_{\mathrm{st}}\left( v \right)}{\sigma_{\mathrm{st}}}$ | σ_st_ denotes the number of shortest paths between nodes s and t, while σ_st_(v) represents the subset of these paths that traverse node v. | Quantifies a node’s control over information flow by counting how often it lies on the shortest paths between other node pairs. |
| Closeness | $C_{C}\left( v \right)=\frac{N-1}{\sum_{s\neq v} d_{\mathrm{sv}}}$ | N is the total number of nodes in the network; d_sv_ is the shortest path distance between nodes s and v. | Indicates how close a node is to all other nodes by measuring the reciprocal of the average shortest path length. |
| Degree | $C_{D}\left( v \right)=k_{v}$ | k_v_ denotes the degree of node v, representing the number of edges directly connected to it. | Measures the immediate connectivity of a node, i.e., the count of its direct neighbors. |
| Eigenvector | $C_{E}\left( v \right)=\frac{1}{\lambda}\sum_{s\in N\left( v \right)} C_{E}\left( s \right)$ | N(v) is the set of neighboring nodes of v; λ is the eigenvalue; C_E_(s) is the eigenvector centrality of neighbors. | Evaluates a node’s influence by considering both its direct connections and the centrality of its neighbors. |
| Harmonic | $C_{H}\left( v \right)=\frac{N-1}{\sum_{s\neq v} \frac{1}{d_{\mathrm{sv}}}}$ | N is the number of nodes in the network;  d_sv_ is the shortest path distance between nodes s and v. The summation excludes v itself. | Measures a node’s accessibility by summing the reciprocal of its distances to all other nodes. |
| Pagerank | $C_{\mathrm{PR}}\left( v \right)=\frac{1-d}{N}+d\sum_{s\in M\left( v \right)} \frac{\mathrm{PR}\left( s \right)}{L\left( s \right)}$ | N is the total number of nodes;  d is the damping factor (commonly 0.85);  PR(s) is the PageRank value of node s;  L(s) is the out-degree of node s | Assesses node importance based on a random walk model, where links from highly ranked nodes contribute more to a node’s score. |
| Strengths | $C_{S}\left( v \right)=\sum_{u\in N\left( v \right)} w_{\mathrm{vu}}$ | w_vu_ denotes the weight of the edge connecting node v and its neighbor u;  N(v) represents the set of all neighboring nodes of v | Quantifies the total weighted connectivity of a node by summing the strengths of all its adjacent edges. |
| DomiRank | $\Gamma=\theta\sigma\left( \sigma A+I_{N\times N} \right)^{-1}AI_{N\times1}$ | A is the N×N adjacency matrix of the network.  θ is the dominance threshold controlling the competitive balance among nodes.  σ is the competition ratio determining the intensity of node competition.  I_N×N_ is the identity matrix with ones on the diagonal and zeros elsewhere.  I_N×1_ is a column vector of ones. | Evaluates the importance of a node by balancing the local (node-level) and mesoscale (structural-level) information in the network. |

# Supplemental Tables

## Table S1. fMRI scanner and image-acquisition protocol information for MCAD

| Site | Field of strength | Brand | Number of head coil channels | Protocol name | Repetition time | Echo time | Flip angle | Field of view | Matrix | Slice number /thickness (gap) | Scan duration |
| --- | --- | --- | --- | --- | --- | --- | --- | --- | --- | --- | --- |
| S01 | 3.0 T | Siemens Skyra | 20 | EPI | 2 s | 30 ms | 90 | 220 × 220 | 64 × 64 | 36 / 3 (0.9) | 8 min |
| S02 | 3.0 T | GE Signa HDx | 8 | EPI | 2 s | 30 ms | 90 | 220 × 220 | 64 × 64 | 30 / 3 (1) | 6 min 40 s |
| S03 | 3.0 T | Siemens Trio Tim | 20 | EPI | 2 s | 25 ms | 90 | 240 × 240 | 64 × 64 | 30 / 3 (1) | 6 min |
| S04 | 3.0 T | Siemens Verio | 8 | EPI | 2 s | 30 ms | 90 | 220 × 220 | 64 × 64 | 36 / 3 (0.99) | 6 min |
| S05 | 3.0 T | Siemens Trio Tim | 12 | EPI | 2 s | 40 ms | 90 | 256 × 256 | 64 × 64 | 28 / 4 (1) | 7 min 58 s |
| S06 | 3.0 T | Siemens Trio Tim | 8 | EPI | 2 s | 30 ms | 90 | 220 × 220 | 64 × 64 | 32 / 3 (0.99) | 6 min |
| S07 | 3.0T | Siemens Skyra | 20 | EPI | 2 s | 30 ms | 90 | 220 × 220 | 64 × 64 | 36 / 3 (0.9) | 8 min |

## Table S2. The statistical results of the classification

Classification results based on eight centralities based on 4% threshold. Other threshold results are shown in Figure S6.

| Centrality | AUC | ACC | SEN | SPE |
| --- | --- | --- | --- | --- |
| DomiRank | 0.7761 | 0.6894 | 0.7341 | 0.6644 |
| Degree | 0.7640 | 0.6759 | 0.6912 | 0.6662 |
| Betweenness | 0.6752 | 0.6087 | 0.7610 | 0.4561 |
| Closeness | 0.7613 | 0.6637 | 0.7085 | 0.6257 |
| Harmonic | 0.7704 | 0.6832 | 0.7394 | 0.6319 |
| Pagerank | 0.7391 | 0.6013 | 0.8454 | 0.2691 |
| Eigenvector | 0.7291 | 0.6639 | 0.6551 | 0.6862 |
| Strengths | 0.7652 | 0.6833 | 0.7116 | 0.6656 |

## Table S3. Detailed statistical results of gene expression

Top 20 clusters with their representative enriched terms (one per cluster). "Count" is the number of genes in the user-provided lists with membership in the given ontology term. "Gene proportion (%)" is the percentage of all of the user-provided genes that are found in the given ontology term (only input genes with at least one ontology term annotation are included in the calculation). "Log10(P)" is the p-value in log base 10. "Log10(q)" is the multi-test adjusted q-value (FDR correction) in log base 10.

| Category | Term | Description | Count | Gene  proportion (%) | Log10(P) | Log10(q) |
| --- | --- | --- | --- | --- | --- | --- |
| GO Biological Processes | GO:0099536 | synaptic signaling | 36 | 7.83 | -13.9 | -9.60 |
| GO Biological Processes | GO:0099537 | trans-synaptic signaling | 34 | 7.89 | -13.3 | -9.30 |
| GO Biological Processes | GO:0098916 | anterograde trans-synaptic signaling | 33 | 7.97 | -13.0 | -9.30 |
| GO Biological Processes | GO:0007268 | chemical synaptic transmission | 33 | 7.97 | -13.0 | -9.30 |
| GO Biological Processes | GO:0050804 | modulation of chemical synaptic transmission | 30 | 6.04 | -8.97 | -5.38 |
| GO Biological Processes | GO:0099177 | regulation of trans-synaptic signaling | 30 | 604 | -8.95 | -5.38 |
| GO Biological Processes | GO:0007507 | heart development | 32 | 5.57 | -8.66 | -5.16 |
| GO Biological Processes | GO:1903530 | regulation of secretion by cell | 31 | 5.61 | -8.46 | -5.07 |
| GO Biological Processes | GO:0034762 | regulation of transmembrane transport | 29 | 5.91 | -8.46 | -5.07 |
| GO Biological Processes | GO:0051046 | regulation of secretion | 32 | 5.27 | -8.08 | -4.73 |
| GO Biological Processes | GO:0007423 | sensory organ development | 31 | 5.33 | -7.94 | -4.64 |
| Reactome Gene Sets | R-HSA-372790 | Signaling by GPCR | 34 | 4.80 | -7.53 | -4.27 |
| GO Biological Processes | GO:0046883 | regulation of hormone secretion | 19 | 7.45 | -7.29 | -4.06 |
| KEGG Pathway | hsa04820 | Cytoskeleton in muscle cells | 18 | 7.76 | -7.21 | -4.01 |
| GO Biological Processes | GO:1904062 | regulation of monoatomic cation transmembrane transport | 21 | 6.65 | -7.14 | -3.97 |
| GO Biological Processes | GO:0010817 | regulation of hormone levels | 28 | 5.18 | -6.97 | -3.83 |
| GO Biological Processes | GO:0000902 | cell morphogenesis | 32 | 4.66 | -6.84 | -3.72 |
| Canonical Pathways | M3008 | NABA ECM GLYCOPROTEINS | 16 | 8.16 | -6.78 | -3.69 |
| GO Biological Processes | GO:0031175 | neuron projection development | 32 | 4.61 | -6.74 | -3.67 |
| Reactome Gene Sets | R-HSA-388396 | GPCR downstream signalling | 30 | 4.73 | -6.59 | -3.55 |

# Supplemental Figures

## Figure S1. Group differences for each center.


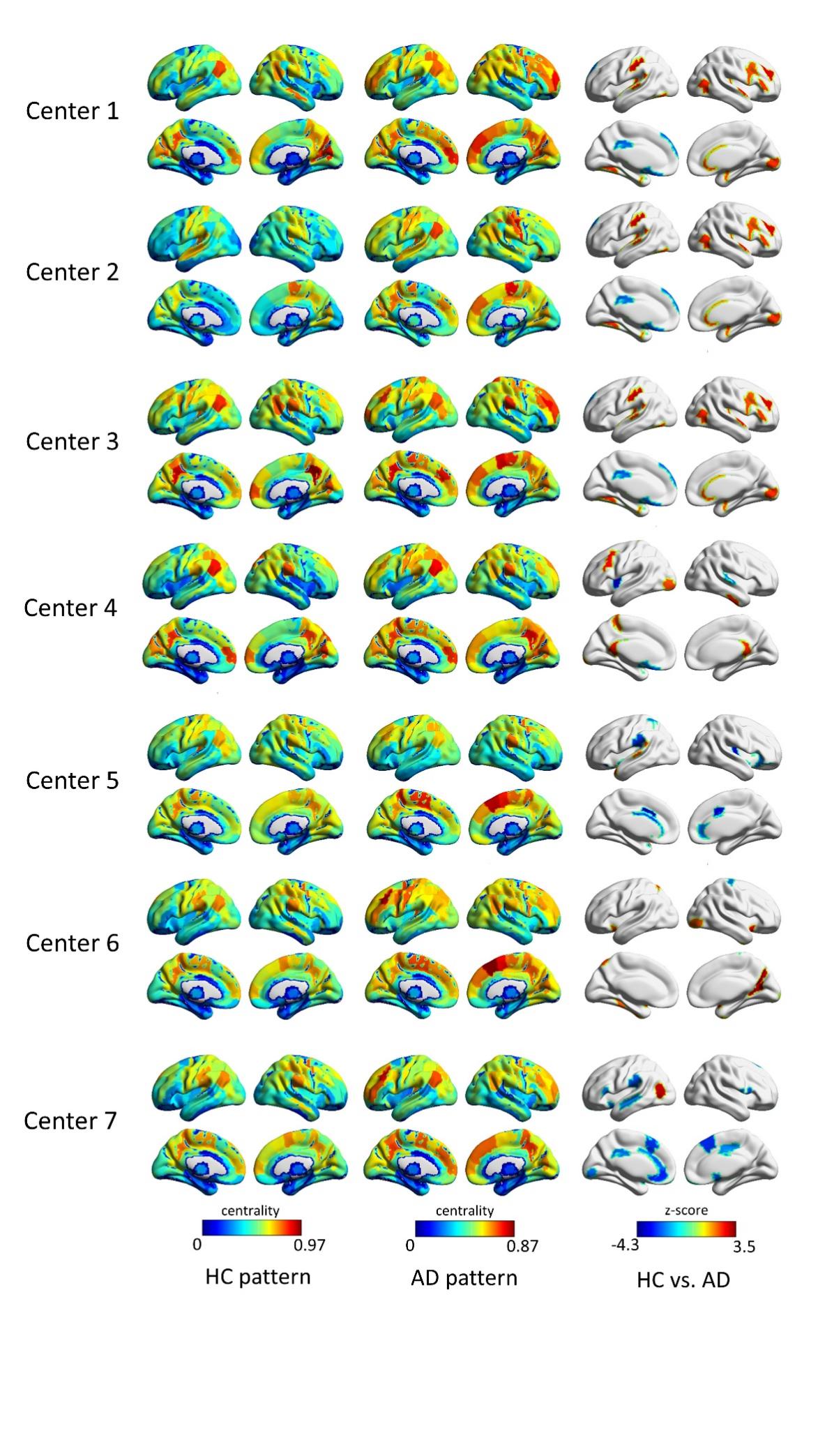


Results from the seven centers demonstrated a high degree of spatial consistency between the HC and AD patterns. The HC group consistently exhibited higher centrality values, predominantly in the parietal regions (including the precuneus, superior parietal lobule, and supramarginal gyrus) as well as portions of the frontal cortex. In contrast, the AD group exhibited a marked reduction in centrality, particularly in the parietal and frontal areas, indicating a progressive decline in hub functions with disease progression. The difference maps further confirmed that regions with significantly reduced centrality in AD were primarily located in the bilateral parietal cortex (superior parietal lobule, supramarginal gyrus, and precuneus) and the frontal cortex.

## Figure S2. Centralities difference and correlation of MMSE.


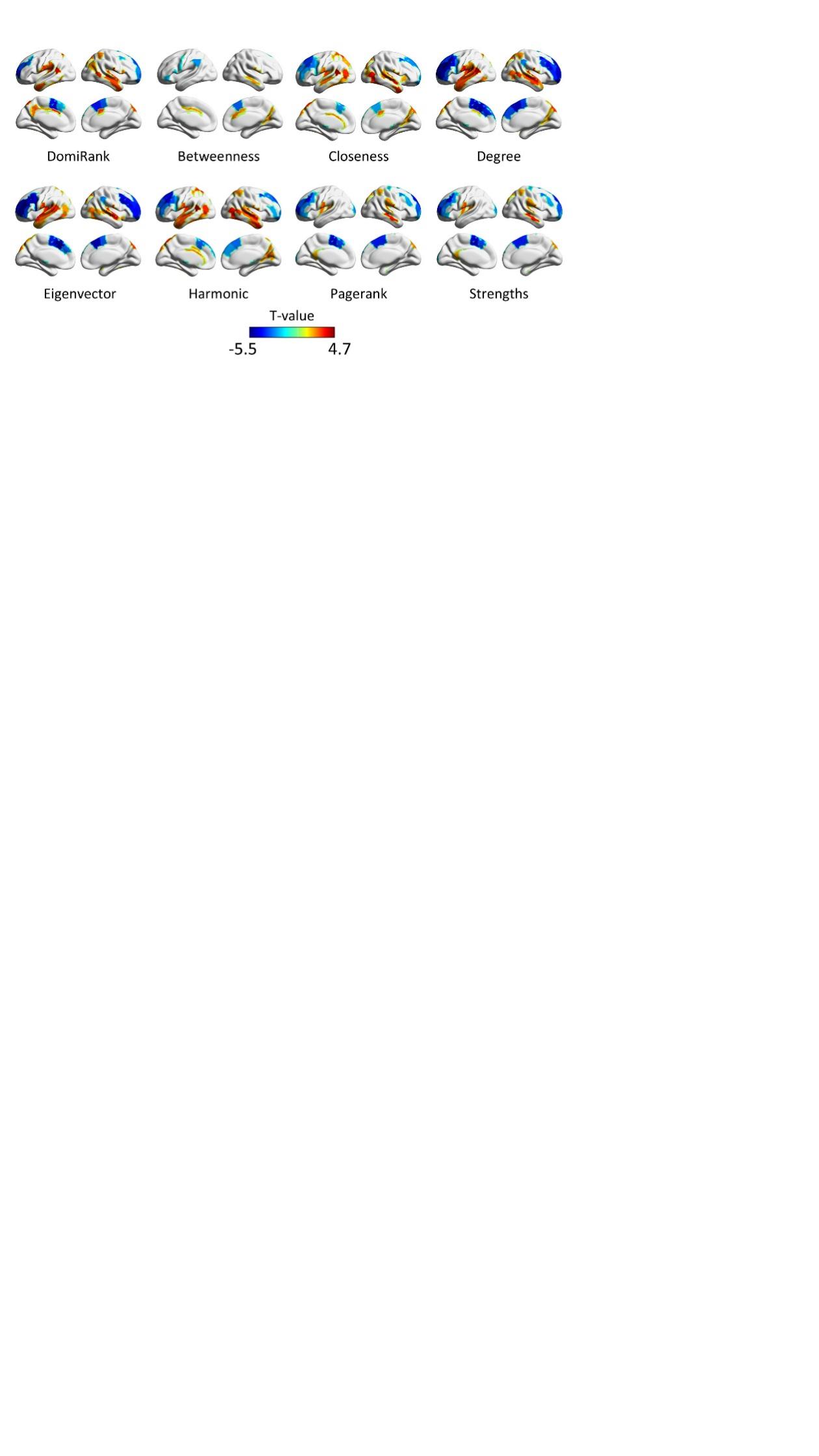


Positive correlations between MMSE scores and eight centralities were primarily observed in the somatomotor network (SMN), other primary sensory networks, and parts of the default mode network (DMN). In contrast, negative correlations were mainly located within the frontoparietal network (FPN) and specific regions of the DMN.

## Figure S3. Correlation between DomiRank centrality and MoCa.


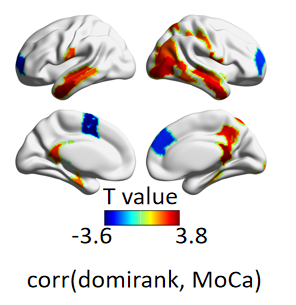


There were 31 regions associated with DomiRank centrality and MoCA scores, as determined by multiple linear regression (*p_FDR_ < 0.05*). The positive correlations were mainly located in the bilateral parietal areas, including parts of the superior parietal lobule and the supramarginal gyrus, as well as portions of the frontal lobe. In contrast, the negative correlations were observed in the medial parietal and posterior cingulate areas.

## Figure S4. Centrality-based attacks based on different threshold.


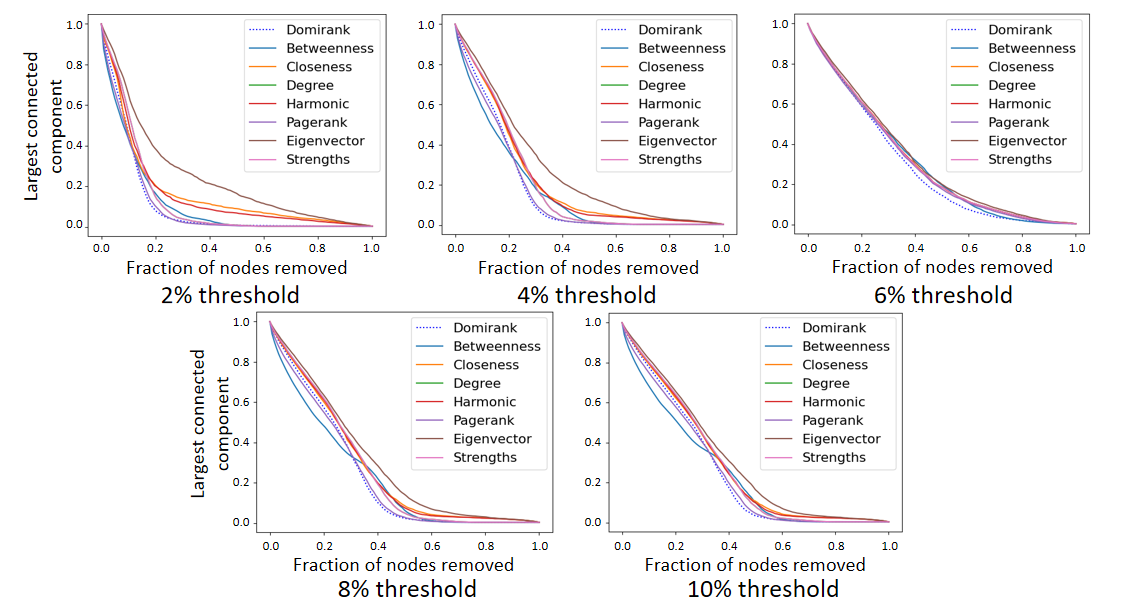


By disrupting the network based on the magnitude of centrality values, it can be observed that DomiRank centrality consistently ranks among the top three centrality metrics across different thresholds at the group level. This further demonstrates that DomiRank centrality holds an advantage in identifying core nodes within the network.

## Figure S5. Classification task based on DomiRank centrality at different thresholds.


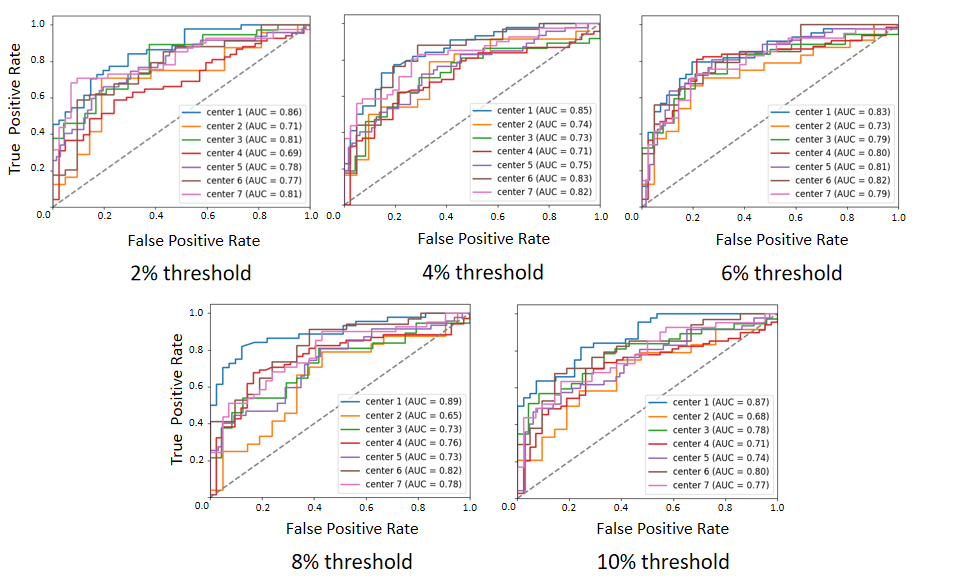


Across all thresholds, DomiRank centrality achieved consistently high classification performance, maintaining an average AUC of approximately 75%–80%, even under cross-site analyses.

## Figure S6. Classification task based on eight centralities at different thresholds.


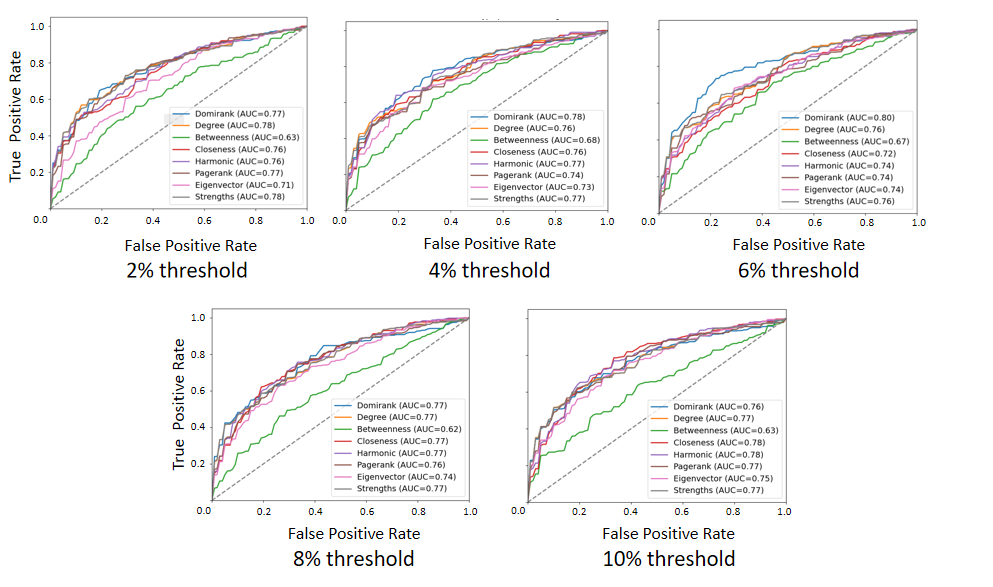


Across all tested thresholds (2%, 4%, 6%, 8%, and 10%), DomiRank centrality consistently ranked among the top-performing metrics and achieved the best classification performance at the 4% and 6% thresholds compared with the other 7 centralities.

## Figure S7. Main results of different datasets.


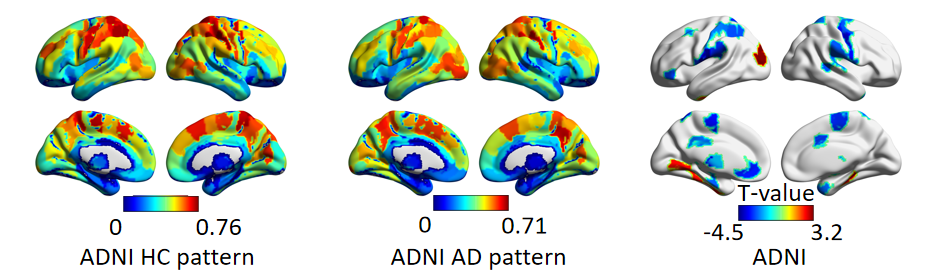


To further verify the robustness of DomiRank centrality on different datasets, we calculated DomiRank centrality on the ADNI dataset. Pearson correlation analysis demonstrated strong consistency between the two datasets, with r = 0.83 for the HC pattern, r = 0.81 for the AD pattern. This indicated that the DomiRank centrality yields stable results across different datasets.

## Figure S8. The effect of different brain atlases on the main result.


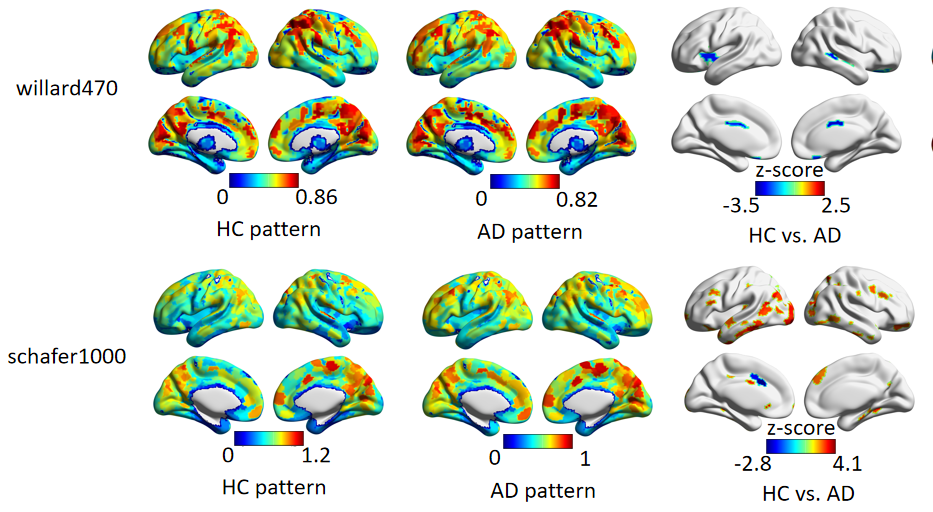


To further evaluate the robustness of DomiRank centrality across different parcellation schemes, we compared the results obtained using the Willard470 and Schafer1000 atlases with those derived from the BN246 atlas. All results were projected back into voxel space to enable direct spatial comparison. For the Willard470 atlas, the similarity with BN246 was r = 0.7782 for the healthy control (HC) pattern, r = 0.7774 for the Alzheimer’s disease (AD) pattern, and r = 0.4982 for the HC vs. AD difference map. In contrast, the Schafer1000 atlas demonstrated higher similarity, with r = 0.8877 for the HC pattern, r = 0.8809 for the AD pattern, and r = 0.5672 for the difference map. Notably, the comparison for Schafer1000 was performed only on cortical surface results.

## **Figure S9.** The effect of the absolute threshold on the main result.


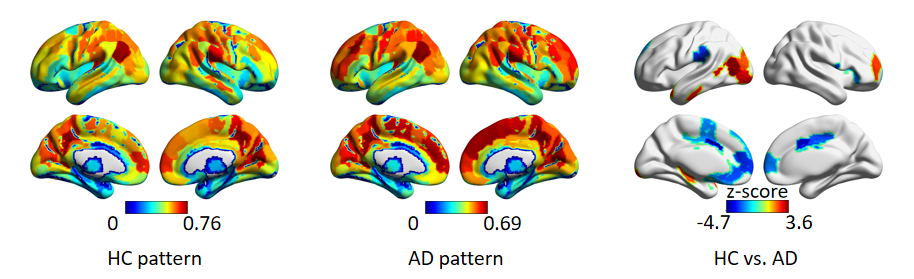


To further assess the robustness of DomiRank centrality under different thresholding strategies, we compared the results obtained using the 4% threshold with those derived from the absolute 4% threshold. Pearson correlation analysis demonstrated strong consistency between the two datasets, with r = 0.9998 for the HC pattern, r = 0.9997 for the AD pattern, and r = 0. 0.9940 for the difference maps. These findings indicate that DomiRank centrality produces highly stable results regardless of the thresholding strategy applied.

## Figure S10. The effect of the length of the time series on the main result.


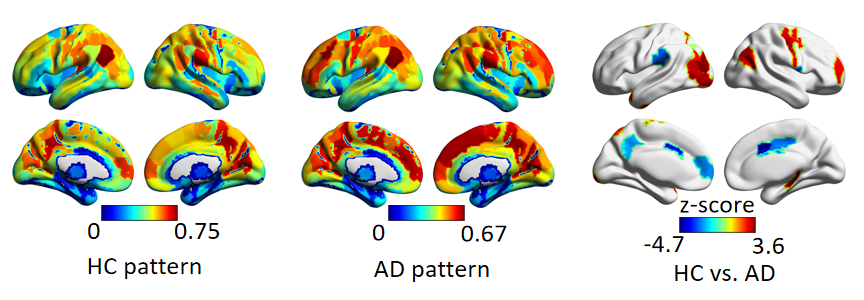


To further evaluate the robustness of DomiRank centrality with respect to different time series lengths, we compared the results obtained using the full-length time series with those derived from a shortened time series (the first 170 frames). Pearson correlation analysis demonstrated strong consistency between the two datasets, with r = 0.9974 for the HC pattern, r = 0.9980 for the AD pattern, and r = 0.9171 for the difference maps. These findings indicate that DomiRank centrality yields highly stable results regardless of the time series length used.

# References

Chen, P., Yao, H., Tijms, B.M., Wang, P., Wang, D., Song, C., Yang, H., Zhang, Z., Zhao, K., Qu, Y., Kang, X., Du, K., Fan, L., Han, T., Yu, C., Zhang, X., Jiang, T., Zhou, Y., Lu, J., Han, Y., Liu, B., Zhou, B., Liu, Y., 2023. Four Distinct Subtypes of Alzheimer's Disease Based on Resting-State Connectivity Biomarkers. Biol Psychiatry 93, 759-769.

Choo, I.H., Lee, D.Y., Youn, J.C., Jhoo, J.H., Kim, K.W., Lee, D.S., Lee, J.S., Woo, J.I., 2007. Topographic patterns of brain functional impairment progression according to clinical severity staging in 116 Alzheimer disease patients: FDG-PET study. Alzheimer Dis Assoc Disord 21, 77-84.

Dozeman, E., van Schaik, D.J., van Marwijk, H.W., Stek, M.L., van der Horst, H.E., Beekman, A.T., 2011. The center for epidemiological studies depression scale (CES-D) is an adequate screening instrument for depressive and anxiety disorders in a very old population living in residential homes. Int J Geriatr Psychiatry 26, 239-246.

Feng, F., Wang, P., Zhao, K., Zhou, B., Yao, H., Meng, Q., Wang, L., Zhang, Z., Ding, Y., Wang, L., An, N., Zhang, X., Liu, Y., 2018. Radiomic Features of Hippocampal Subregions in Alzheimer's Disease and Amnestic Mild Cognitive Impairment. Front Aging Neurosci 10, 290.

Folstein, M.F., Folstein, S.E., McHugh, P.R., 1975. “Mini-mental state”: A practical method for grading the cognitive state of patients for the clinician. Journal of Psychiatric Research 12, 189-198.

Garrison, K.A., Scheinost, D., Finn, E.S., Shen, X., Constable, R.T., 2015. The (in)stability of functional brain network measures across thresholds. NeuroImage 118, 651-661.

Guo, Q., Sun, Y., Yu, P., Hong, Z., Lu, C., 2007. Norm of Auditory Verbal Learning Test in the Normal Aged in China Community. Chinese Journal of Clinical Psychology 15, 132-134.

Guo, Y., Zhang, Z., Zhou, B., Wang, P., Yao, H., Yuan, M., An, N., Dai, H., Wang, L., Zhang, X., Liu, Y., 2014. Grey-matter volume as a potential feature for the classification of Alzheimer's disease and mild cognitive impairment: an exploratory study. Neurosci Bull 30, 477-489.

Hachinski, V.C., Iliff, L.D., Zilhka, E., Du Boulay, G.H., McAllister, V.L., Marshall, J., Russell, R.W., Symon, L., 1975. Cerebral blood flow in dementia. Arch Neurol 32, 632-637.

Hamilton, M., 1960. A rating scale for depression. J Neurol Neurosurg Psychiatry 23, 56-62.

He, X., Qin, W., Liu, Y., Zhang, X., Duan, Y., Song, J., Li, K., Jiang, T., Yu, C., 2014. Abnormal salience network in normal aging and in amnestic mild cognitive impairment and Alzheimer's disease. Hum Brain Mapp 35, 3446-3464.

Jin, D., Wang, P., Zalesky, A., Liu, B., Song, C., Wang, D., Xu, K., Yang, H., Zhang, Z., Yao, H., Zhou, B., Han, T., Zuo, N., Han, Y., Lu, J., Wang, Q., Yu, C., Zhang, X., Zhang, X., Jiang, T., Zhou, Y., Liu, Y., 2020. Grab-AD: Generalizability and reproducibility of altered brain activity and diagnostic classification in Alzheimer's Disease. Hum Brain Mapp 41, 3379-3391.

Lawton, M.P., Brody, E.M., 1969. Assessment of Older People: Self-Maintaining and Instrumental Activities of Daily Living1. The Gerontologist 9, 179-186.

Li, J., Jin, D., Li, A., Liu, B., Song, C., Wang, P., Wang, D., Xu, K., Yang, H., Yao, H., Zhou, B., Bejanin, A., Chetelat, G., Han, T., Lu, J., Wang, Q., Yu, C., Zhang, X., Zhou, Y., Zhang, X., Jiang, T., Liu, Y., Han, Y., 2019. ASAF: altered spontaneous activity fingerprinting in Alzheimer's disease based on multisite fMRI. Science Bulletin 64, 998–1010.

Li, S., Yuan, X., Pu, F., Li, D., Fan, Y., Wu, L., Chao, W., Chen, N., He, Y., Han, Y., 2014. Abnormal changes of multidimensional surface features using multivariate pattern classification in amnestic mild cognitive impairment patients. J Neurosci 34, 10541-10553.

Liu, J., Zhang, X., Yu, C., Duan, Y., Zhuo, J., Cui, Y., Liu, B., Li, K., Jiang, T., Liu, Y., 2016. Impaired Parahippocampus Connectivity in Mild Cognitive Impairment and Alzheimer's Disease. J Alzheimers Dis 49, 1051-1064.

Liu, Y., Yu, C., Zhang, X., Liu, J., Duan, Y., Alexander-Bloch, A.F., Liu, B., Jiang, T., Bullmore, E., 2014. Impaired long distance functional connectivity and weighted network architecture in alzheimer's disease. Cerebral Cortex 24, 1422–1435.

Lu, J., Li, D., Li, F., Zhou, A., Wang, F., Zuo, X., Jia, X.F., Song, H., Jia, J., 2011. Montreal cognitive assessment in detecting cognitive impairment in Chinese elderly individuals: a population-based study. J Geriatr Psychiatry Neurol 24, 184-190.

McKhann, G., Drachman, D., Folstein, M., Katzman, R., Price, D., Stadlan, E.M., 1984. Clinical diagnosis of Alzheimer's disease: report of the NINCDS-ADRDA Work Group under the auspices of Department of Health and Human Services Task Force on Alzheimer's Disease. Neurology 34, 939-944.

McKhann, G.M., Knopman, D.S., Chertkow, H., Hyman, B.T., Jack, C.R., Jr., Kawas, C.H., Klunk, W.E., Koroshetz, W.J., Manly, J.J., Mayeux, R., Mohs, R.C., Morris, J.C., Rossor, M.N., Scheltens, P., Carrillo, M.C., Thies, B., Weintraub, S., Phelps, C.H., 2011. The diagnosis of dementia due to Alzheimer's disease: recommendations from the National Institute on Aging-Alzheimer's Association workgroups on diagnostic guidelines for Alzheimer's disease. Alzheimers Dement 7, 263-269.

Morris, J.C., 1993. The Clinical Dementia Rating (CDR): current version and scoring rules. Neurology 43, 2412-2414.

Petersen, R.C., 2004. Mild cognitive impairment as a diagnostic entity. J Intern Med 256, 183-194.

Petersen, R.C., Doody, R., Kurz, A., Mohs, R.C., Morris, J.C., Rabins, P.V., Ritchie, K., Rossor, M., Thal, L., Winblad, B., 2001. Current concepts in mild cognitive impairment. Arch Neurol 58, 1985-1992.

Petersen, R.C., Smith, G.E., Waring, S.C., Ivnik, R.J., Tangalos, E.G., Kokmen, E., 1999. Mild cognitive impairment: clinical characterization and outcome. Arch Neurol 56, 303-308.

Qu, Y., Wang, P., Liu, B., Song, C., Wang, D., Yang, H., Zhang, Z., Chen, P., Kang, X., Du, K., Yao, H., Zhou, B., Han, T., Zuo, N., Han, Y., Lu, J., Yu, C., Zhang, X., Jiang, T., Zhou, Y., Liu, Y., 2021. AI4AD: Artificial intelligence analysis for Alzheimer's disease classification based on a multisite DTI database. Brain Disorders 1, 100005.

Shi, Y., Wang, Z., Chen, P., Cheng, P., Zhao, K., Zhang, H., Shu, H., Gu, L., Gao, L., Wang, Q., Zhang, H., Xie, C., Liu, Y., Zhang, Z., Alzheimer's Disease Neuroimaging, I., 2023. Episodic Memory-Related Imaging Features as Valuable Biomarkers for the Diagnosis of Alzheimer's Disease: A Multicenter Study Based on Machine Learning. Biol Psychiatry Cogn Neurosci Neuroimaging 8, 171-180.

Song, J., Qin, W., Liu, Y., Duan, Y., Liu, J., He, X., Li, K., Zhang, X., Jiang, T., Yu, C., 2013. Aberrant functional organization within and between resting-state networks in AD. PLoS One 8, e63727.

Sun, Y., Wang, P., Zhao, K., Chen, P., Qu, Y., Li, Z., Zhong, S., Zhou, B., Lu, J., Zhang, X., Wang, D., Han, Y., Yao, H., Liu, Y., 2024. Structure-function coupling reveals the brain hierarchical structure dysfunction in Alzheimer's disease: A multicenter study. Alzheimers Dement 20, 6305-6315.

van den Heuvel, M.P., de Lange, S.C., Zalesky, A., Seguin, C., Yeo, B.T.T., Schmidt, R., 2017. Proportional thresholding in resting-state fMRI functional connectivity networks and consequences for patient-control connectome studies: Issues and recommendations. NeuroImage 152, 437-449.

Wang, P., Zhou, B., Yao, H., Zhan, Y., Zhang, Z., Cui, Y., Xu, K., Ma, J., Wang, L., An, N., Zhang, X., Liu, Y., Jiang, T., 2015. Aberrant intra- and inter-network connectivity architectures in Alzheimer's disease and mild cognitive impairment. Sci Rep 5, 14824.

Wu, Y., Zhang, Y., Liu, Y., Liu, J., Duan, Y., Wei, X., Zhuo, J., Li, K., Zhang, X., Yu, C., Wang, J., Jiang, T., 2016. Distinct Changes in Functional Connectivity in Posteromedial Cortex Subregions during the Progress of Alzheimer's Disease. Front Neuroanat 10, 41.

Yan, T., Wang, W., Yang, L., Chen, K., Chen, R., Han, Y., 2018. Rich club disturbances of the human connectome from subjective cognitive decline to Alzheimer's disease. Theranostics 8, 3237-3255.

Yao, H., Zhou, B., Zhang, Z., Wang, P., Guo, Y., Shang, Y., Wang, L., Zhang, X., An, N., Liu, Y., Alzheimer's Disease Neuroimaging, I., 2014. Longitudinal alteration of amygdalar functional connectivity in mild cognitive impairment subjects revealed by resting-state FMRI. Brain Connect 4, 361-370.

Yesavage, J.A., Brink, T.L., Rose, T.L., Lum, O., Huang, V., Adey, M., Leirer, V.O., 1982. Development and validation of a geriatric depression screening scale: a preliminary report. J Psychiatr Res 17, 37-49.

Zhang, Z., Liu, Y., Zhou, B., Zheng, J., Yao, H., An, N., Wang, P., Guo, Y., Dai, H., Wang, L., Shu, S., Zhang, X., Jiang, T., 2014. Altered functional connectivity of the marginal division in Alzheimer's disease. Curr Alzheimer Res 11, 145-155.

Zhou, B., Yao, H., Wang, P., Zhang, Z., Zhan, Y., Ma, J., Xu, K., Wang, L., An, N., Liu, Y., Zhang, X., 2015. Aberrant Functional Connectivity Architecture in Alzheimer's Disease and Mild Cognitive Impairment: A Whole-Brain, Data-Driven Analysis. Biomed Res Int 2015, 495375.
